# Lipoxins in the Nervous System: Brighter Prospects for Neuroprotection

**DOI:** 10.3389/fphar.2022.781889

**Published:** 2022-01-26

**Authors:** Jiayu Zhang, Zhe Li, Mingyue Fan, Wei Jin

**Affiliations:** ^1^ Graduate School of Hebei Medical University, Shijiazhuang, China; ^2^ Department of Neurology, Hebei General Hospital, Shijiazhuang, China

**Keywords:** lipoxins, neuroprotection, neurological diseases, resolution of inflammation, anti-oxidation

## Abstract

Lipoxins (LXs) are generated from arachidonic acid and are involved in the resolution of inflammation and confer protection in a variety of pathological processes. In the nervous system, LXs exert an array of protective effects against neurological diseases, including ischemic or hemorrhagic stroke, neonatal hypoxia-ischemia encephalopathy, brain and spinal cord injury, Alzheimer’s disease, multiple sclerosis, and neuropathic pain. Lipoxin administration is a potential therapeutic strategy in neurological diseases due to its notable efficiency and unique superiority regarding safety. Here, we provide an overview of LXs in terms of their synthesis, signaling pathways and neuroprotective evidence. Overall, we believe that, along with advances in lipoxin-related drug design, LXs will bring brighter prospects for neuroprotection.

## 1 Introduction

Resolution is a crucial stage of the inflammatory response, which is necessary to limit excessive tissue injury, minimize the development of chronic inflammation and re-establish homeostasis. During the process, specialized pro-resolving mediators (SPMs) with anti-inflammatory actions, including lipoxins (LXs), resolvins, protectins, and maresins, may be generated (Serhan, 2014). Among these endogenous local mediators, LXs, a class of arachidonate (arachidonic acid, AA)-derived eicosanoids, are the first to be recognized for functioning as “braking signals” in inflammation (Serhan et al., 1984). They are typically generated by lipoxygenase (LOX) interactions in a biosynthetic pathway known as transcellular biosynthesis (Serhan et al., 1994). In the past few decades, the actions of LXs in inflammation have been gradually determined. They can decrease the production of proinflammatory mediators, including interleukin (IL)-1, IL-6 and tumor necrosis factor (TNF)-α; facilitate the release of anti-inflammatory cytokines such as transforming growth factor-β1, IL-10 and prostaglandin E_2_ (PGE_2_); and consequently promote the resolution of inflammation. Additionally, they are found to inhibit neutrophil chemotaxis and infiltration, promote the phagocytic clearance of apoptotic cells by macrophages, and stimulate the accumulation of a nonphlogistic type of monocytes/macrophages (Lawrence et al., 2002; Maderna and Godson, 2009). Owing to their wide spectrum of anti-inflammatory and pro-resolving properties, a multitude of studies have investigated the potential protective effects of LXs on a variety of diseases and the underlying mechanism.

It has become widely appreciated that, in addition to classic infectious diseases such as encephalitis, excessive inflammation also occurs in the pathogenesis of many other neurologicaldiseases including stroke, neurotrauma, and neurodegenerative diseases (Ransohoff, 2016; Devanney et al., 2020; Mészáros et al., 2020). Moderate neuroinflammation orchestrated by microglia, macrophages and lymphocytes is a beneficial response to foreign challenge or tissue injury, which can ultimately lead to the restoration of tissue structure and function. Generally, this type of inflammatory response is self-limiting under the strict control of endogenous mechanisms (Lawrence et al., 2002). However, prolonged inflammation can override the beneficial actions and contribute to the disease course. Taking ischemic stroke as an example, microglia-associated neuroinflammation can play an important role in isolating damaged brain tissue and clearing dead cell debris in the central nervous system (CNS), whereas the vast release of proinflammatory cytokines can lead to secondary brain tissue injury and cause poor functional recovery (Hanisch and Kettenmann, 2007). Thus, targeting the resolution of inflammation has become a promising therapeutic strategy for the treatment of neurological diseases, and LXs have drawn scientists’ attention.

Interestingly, in addition to their involvement in the regulation of inflammation, LXs have also been found to have antioxidative, antiapoptotic, autophagy-moderating actions (Wu et al., 2012c; Jin et al., 2014; Jia et al., 2015; Prieto et al., 2015). In this review, we aimed to summarize the current knowledge about the many effects that LXs have on the nervous system and to discuss their roles in different CNS cell types, as well as their therapeutic potential for neurological diseases.

## 2 The Synthesis of Lipoxins

Endogenous LXs can be categorized into two types: native LXs composed of lipoxin A_4_ (5*S*,6*R*,15*S*-trihydroxy-7,9,13-*trans*-11-*cis*-eicosatetraenoic acid, LXA_4_) and lipoxin B_4_ (5*S*,14*R*,15*S*-trihydroxy-6,10,12-*trans*-8-*cis* eicosatetraenoic acid, LXB_4_) and aspirin-triggered lipoxins (ATLs), including aspirin-triggered lipoxin A_4_ (15-epi-LXA_4_, ATLA_4_) and aspirin-triggered lipoxin B_4_ (15-epi-LXB_4_, ATLB_4_). Compared to native LXs, ATLs are more resistant to metabolic inactivation and have an enhanced ability to evoke bioactions.

Native LXA_4_ and LXB_4_ are positional isomers typically generated from AA mediated by LOXs. There are two main pathways of native LX biosynthesis in human cells and tissues. One way comprises sequential lipoxygenation of AA by 15-LOX in epithelial cells and monocytes and by 5-LOX in neutrophils. In this pathway, not only are LXs synthesized, but leukotriene (LT) formation is also reduced (Serhan et al., 1984). The other involves the conversion of LTA_4_, the 5-LOX epoxide product, to LXA_4_ or LXB_4_ by the LOX-synthetase activity of 12-LOX in platelets, which occurs when platelets adhere to neutrophils (Serhan and Sheppard, 1990). For ATL, aspirin can acetylate cyclooxygenase-2 (COX-2) and switch its catalytic activity from generating the intermediate for prostaglandins (PGs) and thromboxanes (TXs) to an *R*-LOX action, thus producing 15*R*-hydroxyeicosatetraenoic acid (15*R*-HETE). Then, the product is rapidly converted to ATL by 5-LOX (Clària and Serhan, 1995).

Actually, aside from aspirin, several drugs can induce the synthesis of LXA_4_. Pioglitazone and atorvastatin have been reported to increase myocardial levels of 15-epi-LXA_4_ produced by both COX-2 and 5-LOX (Birnbaum et al., 2006). Rosiglitazone can switch the generation of proinflammatory LTB4 to LXA_4_ *via de novo* synthesis of 5-LOX, thus contributing to neuroprotection in experimental stroke (Sobrado et al., 2009).

LXs have been identified in brain tissues and cerebrospinal fluid (CSF), but the specific cell types responsible for generating and secreting LXs have not been illustrated explicitly. In addition to neutrophils, immune cells and endothelial cells (Ke et al., 2017), both microglia and astrocytes have shown the capacity to produce LXs. Immunohistochemical analysis of human hippocampal tissue revealed the localization of 15-LOX-2 in both astrocytes and microglia but not in neurons (Wang et al., 2015). Likewise, another key enzyme involved in LX synthesis, 5-LOX, and its activating protein (FLAP) are also expressed in human microglia (Klegeris and McGeer, 2003). It has been confirmed that human microglia can release LXA_4_ after lipopolysaccharide (LPS)- treatment (Zhu et al., 2015). In the inner retina, astrocytes are able to synthesize LXA_4_ and LXB_4_ to participate in neuroprotection (Livne-Bar et al., 2017). At present, it is not clear which cell types in the CNS are the predominant source of LXs, but single-cell sequencing techniques coupled with lipidomics may be of help to address this problem in the future.

To increase the half-life of LXs, a range of stable, biologically active analogs have been designed and played an important role in studying actions of LXs. They were proven to be as potent as endogenous LXs in a series of *in vitro* and *in vivo* animal models, but only LXA_4_ methyl ester (LXA_4_ ME) and BML-111 (5*S*,6*R*,7-trihydroxyheptanoic acid methyl ester) were tested in the nervous system. Besides, since reliable commercial sources of LXB_4_ have only recently become available, most experiments on LXs in the CNS or their roles in neuroprotection are performed using LXA_4_, ATL and their analogs. Consequently, there is limited knowledge about LXB_4_.

## 3 The Lipoxin Receptor and Signaling Pathway

The actions of LXA_4_ and ATL are primarily mediated by a distinct G protein-coupled receptor (GPR) of the formyl peptide receptor superfamily (FPR). In the course of receptor identification, several different names have been used, including formyl peptide receptor 2 (FPR2), formyl peptide receptor-like 1 (FPRL1), LXA_4_ receptors (LXA_4_R), and ALX (Ye et al., 2009). According to the International Union of Basic and Clinical Pharmacology-recommended nomenclature (Ye et al., 2009), we use the term FPR2/ALX to refer to the receptor in this article regardless of species. FPR2/ALX is expressed in several types of leukocytes, including neutrophils, monocytes/macrophages and activated T cells (Cattaneo et al., 2013). Recently, the expression of FPR2/ALX has also been investigated in brain cells, but the conclusions remain controversial. In animal experiments, a study using double immunofluorescence and western blotting found that, in the rat brain, FPR2/ALX was highly expressed in neurons, moderately expressed in microglia, and not expressed in astrocytes (Liu G.J. et al., 2020). However, FPR2/ALX was detected to be expressed in both primary astrocytes and microglia in a rat meningitis model by using reverse transcription-polymerase chain reaction (RT-PCR) and immunofluorescence (Braun et al., 2011; Abdelmoaty et al., 2013). In humans, the first evidence of FPR expression was reported in 1998 *via* immunocytochemistry (ICC) (Becker et al., 1998). Research detected FPR in brain and spinal cord sections and found positive results in neurons, astrocytes and Schwann cells but negative results in oligodendrocytes and microglia. Inconsequently, another study in 2015 revealed FPR2/ALX expression in both astrocytes and microglia through immunohistochemistry in human hippocampal tissue (Wang et al., 2015). Moreover, neural stem cells (NSCs) can also express FPR2/ALX, which has been confirmed by ICC, RT-PCR and western blotting in rodent pups (Wang et al., 2016). In our view, FPR2/ALX has a wide distribution in the nervous system, and the different results for FPR2/ALX localization may be attributed to the way it was detected. When using ICC or immunohistochemistry, the high expression in neurons may cover up the expression in other cells, causing the absence of observations. A study using RT-PCR indicated that FPR2/ALX mRNA expression was greatest in the brainstem, followed by the spinal cord, thalamus/hypothalamus, cerebral neocortex, hippocampus, cerebellum and striatum (Ho et al., 2018). Therefore, another factor that cannot be ignored is that the expression level of FPR2/ALX varies in different regions of the brain and spinal cord.

The expression of FPR2/ALX usually increases in pathological conditions. In a rat subarachnoid hemorrhage (SAH) model, FPR2/ALX expression was significantly increased and maintained from 24 h to approximately 3 days (Guo et al., 2016; Liu G.J. et al., 2020). Compared to the control brain, a higher FPR2/ALX level was also detected in Alzheimer’s disease (AD) (Wang et al., 2015). Moreover, FPR2/ALX has also been observed to be altered in the spinal cord after peripheral inflammation (Abdelmoaty et al., 2013). FPR2/ALX is a versatile receptor that can bind to a variety of ligands and exert different functions, including both proinflammatory and pro-resolving functions (Cattaneo et al., 2013). It has not yet been ascertained whether the increase in FPR2/ALX triggers inflammatory damage or acts as an endogenous compensatory outcome for the reduced SPMs in the pathological brain to perform neuroprotection. Tylek et al. (2021) introduced the dual actions of FPR2/ALX on inflammatory response regulation in the brain and explained them by the concept of biased agonism. Interestingly, LXs did not show “dual-faced” effects on FPR2/ALX and have been always acting as anti-inflammatory mediators.

**FIGURE 1:**
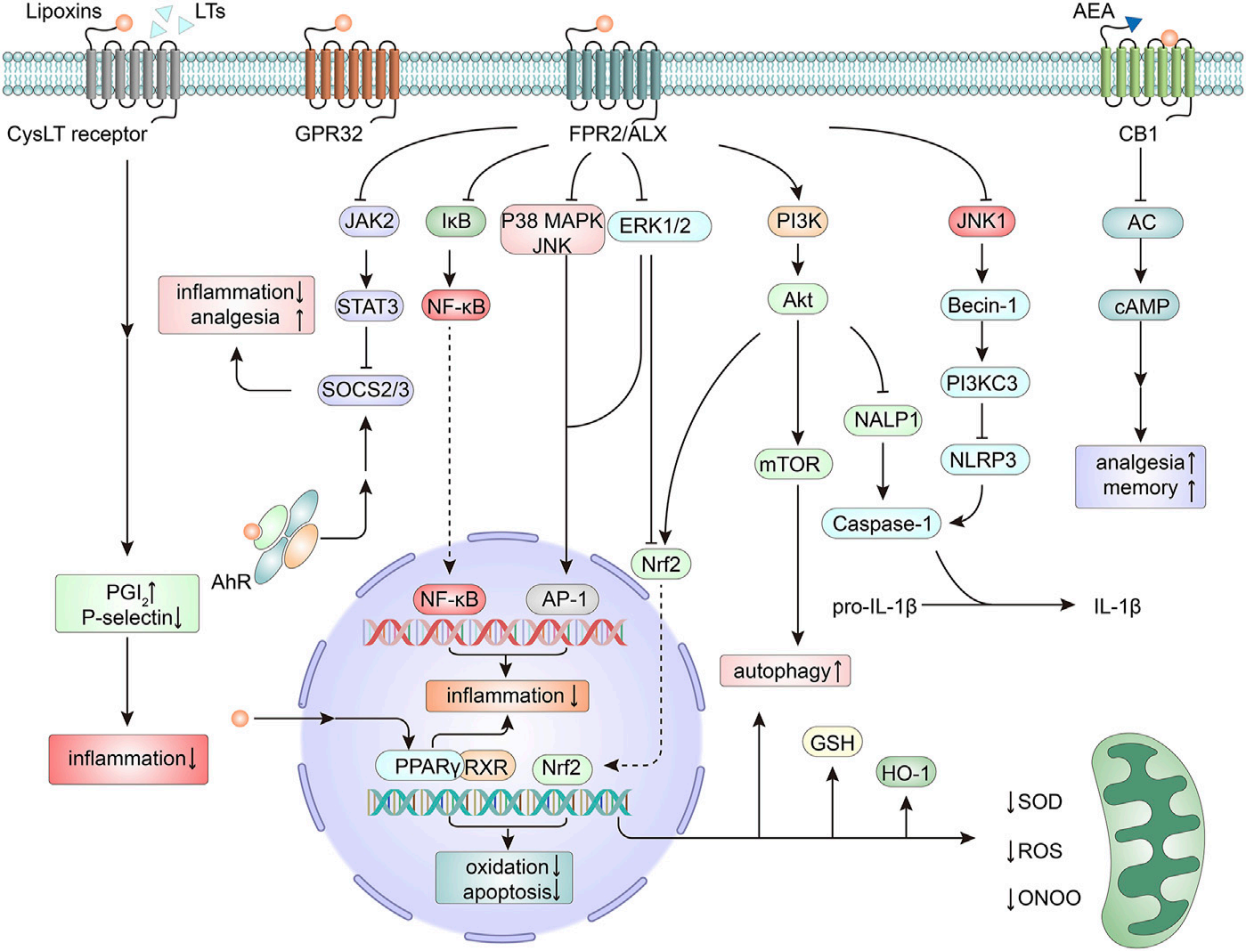
Mechanisms of neuroprotection by lipoxins (LXs). The actions of LXs are mainly mediated by the activation of the formyl peptide receptor 2/LXA_4_ receptor (FPR2/ALX). Downstream from the activation of FPR2/ALX, several signaling pathways are triggered, thus modulating the expression of genes and proteins related to inflammation (Machado et al., 2006; Wang et al., 2011; Wang et al., 2014), apoptosis (Zhu et al., 2020), oxidation (Wu et al., 2012c; Jin et al., 2014), autophagy (Prieto et al., 2015), and pain signaling (Wang et al., 2014). As antagonists of cysteinyl leukotriene (CysLT) receptors, LXs compete for binding sites with leukotrienes (LTs) and mediate an anti-inflammatory action (Norel and Brink, 2004). LXs can bind to G protein-coupled receptor 32 (GPR32) (Zhu et al., 2016) and act as an endogenous allosteric enhancer of the cannabinoid 1 (CB1) receptor (Pamplona et al., 2012). LXs can also exert agonistic action on peroxisome proliferator-activated receptor gamma (PPARγ) (Sobrado et al., 2009), thereby mitigating inflammation and neutralizing oxidative stress. In addition, by activating the nuclear receptor aryl hydrocarbon receptor (AhR), LXs promote the expression of suppressor of cytokine signaling 2 (SOCS2), thus exerting effects on anti-inflammation and analgesia (Schaldach et al., 1999; Machado et al., 2006). AC, adenylate cyclase; AEA, anandamide; Akt, protein kinase B, PKB; AP-1, activating protein-1; cAMP, cyclic adenosine monophosphate; ERK, extracellular signal regulated kinase; GSH, glutathione; HO-1, heme oxygenase-1; IκB, inhibitor κB; JAK2, Janus kinase 2; JNK, c-Jun N-terminal kinase; MAPK, mitogen-activated protein kinase; mTOR, mammalian target of rapamycin; NALP1, NAcht leucine-rich-repeat protein 1; NF-κB, nuclear factor kappa B; Nrf2, nuclear factor erythroid 2-related factor 2; ONOO, peroxynitrite; PG, prostaglandin; PI3K, phosphoinositide-3-kinase; ROS, reactive oxygen species; RXR, retinoid X receptor; SOD, superoxide dismutase; STAT3, signal transducer and activator of transcription 3.

Through FPR2/ALX, LXA_4_ blocks the mitogen-activated protein kinase (MAPK) pathway and attenuates nuclear factor kappa B (NF-κB) activation (Wang et al., 2011), which are two pathways that promote inflammation and neurodegeneration. ATL also depresses the Janus kinase 2 (JAK2)/signal transducer and activator of transcription 3 (STAT3) signaling pathway and triggers the expression of suppressor of cytokine signaling-2/3. Consequently, neuroinflammation and the induction of neuropathic pain are suppressed (Machado et al., 2006; Wang et al., 2014). Moreover, ATL has been shown to activate the protein kinase B (Akt) pathway (Lu et al., 2018), interact with nuclear factor erythroid 2-related factor 2 (Nrf2) and its downstream antioxidant enzymes (Jin et al., 2014), and abrogate nicotinamide adenine dinucleotide phosphate (NADPH) oxidase-dependent reactive oxygen species (ROS) generation (Wu et al., 2012c). In these ways, activation of FPR2/ALX exerts neuroprotection (Figure 1). LXA_4_ can also induce microtubule-associated protein 1 light chain 3 (MAP1LC3)-II from MAP1LC3-I and the degradation of sequestosome 1 as well as the formation of MAP1LC3C^+^ autophagosomes, which modulate apoptosis and autophagy in inflammation. The effect may be related to the activation of MAPK1 and the Nrf2 pathways (Prieto et al., 2015). Furthermore, FPR2/ALX is also the receptor for axonal or dendritic outgrowth (Ho et al., 2018) and the contributor to the migration and differentiation of NSCs (Wang et al., 2016), suggesting its important roles in cell proliferation and differentiation.

LXA_4_ and ATL can also bind to other receptors (Figure 1). LXA_4_ can inhibit the cysteinyl leukotriene receptor in vascular endothelial cells (Norel and Brink, 2004) and activate the aryl hydrocarbon receptor in dendritic cells (Schaldach et al., 1999; Machado et al., 2006), thus mediating anti-inflammatory actions. In the CNS, LXA_4_ can bind to GPR32, which was discovered in differentiated neuroblastoma cells (Zhu et al., 2016). LXA_4_ is also an endogenous allosteric enhancer of the cannabinoid 1 receptor, exerting cannabimimetic effects in the brain (Pamplona et al., 2012). Peroxisome proliferator-activated receptor gamma (PPARγ) has been illustrated to serve as a master gatekeeper of cytoprotective stress responses (Cai et al., 2018). LXA_4_ can act as an agonist of PPARγ, thereby mitigating inflammation and neutralizing oxidative stress (Sobrado et al., 2009).

As a positional isomer of LXA_4_, LXB_4_ carries alcohol groups at the carbon 5S, 14R, and 15S positions instead of the C-5S, 6R, and 15S positions presented in LXA_4_. Although LXB_4_ shares several similar bioactivities with LXA_4_ (Lefer et al., 1988; Takano et al., 1998; Ariel et al., 2003), it has shown differences from LXA_4_ in many ways. In terms of actions, LXB_4_ enhances human memory B cell antibody production, while LXA_4_ confers exactly the reverse effect (Kim et al., 2018). In addition, the generation of LXB_4_ is regulated by NLR family pyrin domain containing 3 (NLRP3) inflammasome activity (Lee et al., 2017), and the ω-oxidation products of LXB_4_ are equipotent with the parent molecule in polymorphonuclear leukocytes (Maddox and Serhan, 1996), which are not seen in LXA_4_. More interestingly, distinct from LXA_4_ signaling, LXB_4_ does not bind to ALXR (Fiore et al., 1994) and does not induce an increase in cytosolic calcium as a component of the signal transduction events following monocyte interaction (Romano et al., 1996). In the one and only study on LXB_4_ neuroprotection, it demonstrated more potent protection than LXA_4,_ and the actions were independent of FPR2/ALX and resolvin D2 receptor (GPR18) (Livne-Bar et al., 2017). With respect to its possible remarkable neuroprotective role, LXB_4_ and its downstream signaling are worth further investigation.

## 4 Protective Effects of Lipoxins in Central Nervous System Cells

**FIGURE 2:**
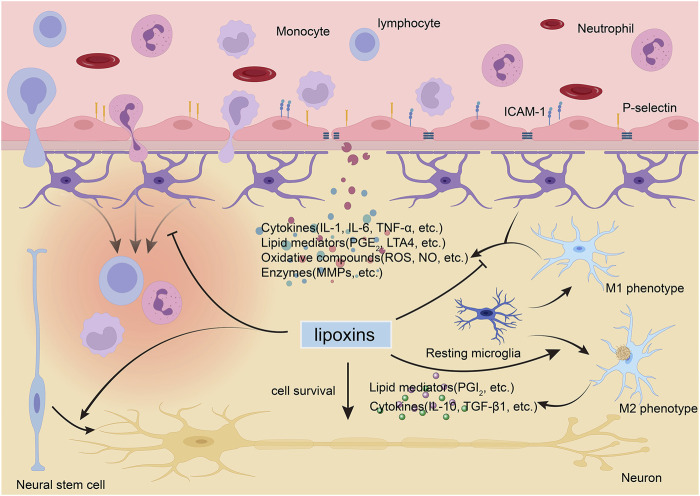
Lipoxins (LXs) exert protective and modulatory actions in the brain. During neurological diseases, the blood-brain barrier (BBB) is disturbed and allows circulating immune cells and proteins to enter the brain. LXs can inhibit the activation and migration of immune cells (Liu et al., 2019), modulate activated endothelial leukocyte interactions (Smith et al., 2015), and maintain the integrity of the BBB by suppressing the expression of proinflammatory mediators and matrix metallopeptidases (MMPs) (Wu et al., 2012b; Hawkins et al., 2014). LXs can modulate glial cell activity to block harmful cytokine release (Wang et al., 2011; Wu et al., 2012a; Yao et al., 2014) and switch activated microglia to the anti-inflammatory, tissue-repairing M2 phenotype instead of the proinflammatory, tissue-damaging M1 phenotype (Taetzsch et al., 2015). LXs can also promote the survival of neurons (Zhu et al., 2020) and inducted the differentiation of neural stem cell to neurons (Wada et al., 2006). ICAM, intercellular cell adhesion molecule-1; IL, interleukin; LT, leukotriene; NO, nitric oxide; PG, prostaglandin; ROS, reactive oxygen species; TGF-β1, transforming growth factor β1; TNF, tumor necrosis factor.

The roles of LXs have been assessed in different CNS cell populations exposed to various stimuli. Herein, we specifically focus upon evidence for the effects of LXs in different CNS cell types, involving not only protection but also modulation (Figure 2).

### 4.1 Neural Stem Cells

NSCs can proliferate, migrate and differentiate into neurons, astrocytes and/or oligodendrocytes. During the pathological process, NSCs can proliferate, migrate to lesions and rebuild the damaged neuronal network in response to extracellular signal changes. It was reported that stable analogs of LXA_4_ and ATLA_4_ could directly regulate the growth of NSCs isolated from embryonic mouse brains by improving growth-related gene expression, including epidermal growth factor receptor, cyclin E, p27, and caspase 8 (Wada et al., 2006). Another study demonstrated that FPR2/ALX detected in NSCs can promote NSC migration through F-actin polymerization and skew NSC differentiation to neurons, implying that LXs may serve as candidates for the treatment of brain or spinal cord injury (Wang et al., 2016).

### 4.2 Neurons

The protective effects of LXs on neurons have also been extensively studied. LXs can reduce neuronal death in response to a variety of stimuli, including staurosporine, glutamate, paraquat, serum deprivation and oxygen-glucose deprivation (Zhu et al., 2016; Livne-Bar et al., 2017; Zhu et al., 2020), which has been explained to be related to their anti-inflammatory, anti-apoptotic and antioxidative effects (Zhu et al., 2016; Zhu et al., 2020). Furthermore, FPR2/ALX mediated axonal and dendritic outgrowth, suggesting that LXs may promote neuronal repair by activating FPR2/ALX (Ho et al., 2018).

### 4.3 Microglia

Microglia are the resident immune cells of the CNS. They are activated in response to pathological insults and harmful stimuli, a process termed polarization. Activated microglia have been largely classified into two phenotypes, namely, classically activated (M1, proinflammatory) or alternatively activated (M2, anti-inflammatory) microglia. Proinflammatory microglia produce inflammatory mediators and exert detrimental effects. Conversely, anti-inflammatory microglia phagocytose cell fragments, dampen the inflammatory response and promote tissue repair. A study demonstrated that proinflammatory microglia could downregulate their capacity to produce LXA_4_, further worsening the imbalance between proinflammation and anti-inflammation (Feng et al., 2017). ATL attenuates LPS-induced proinflammatory responses and the production of nitric oxide by inhibiting the activation of NF-κB and MAPKs *via* FPR2/ALX in BV-2 microglial cells (Wang et al., 2011). ATL could also abrogate NADPH oxidase-mediated ROS generation, subsequently inhibiting oxidase activation (Wu et al., 2012c) and M1 activation in microglia (Taetzsch et al., 2015). Recently, studies have demonstrated that LXs regulated microglial activation through the Notch signaling pathway (Wu et al., 2019b; Li et al., 2021), working as a repressor of inflammatory reactions in the brain. In fact, the supposed dichotomy between M1 and M2 phenotypes is oversimplified. Recent transcriptomics and proteomics studies have identified a multitude of activated microglial phenotypes in diverse disease stages (Beaino et al., 2021). To boost the correct microglial phenotype, it is necessary to further study the role and the underlying mechanism of LXs in regulating microglial activation, especially *in vivo*.

The effects of LXs on macrophages in the resolution of inflammation have been elucidated. LXs could mediate macrophage recruitment, improve the nonphlogistic phagocytosis of apoptotic neutrophils by macrophages (Godson et al., 2000), and increase macrophage survival *via* inhibition of apoptosis (Prieto et al., 2010) and regulation of autophagy (Prieto et al., 2015). In addition, LXs could also shift the macrophage phenotypic profile to an M2 state (Vasconcelos et al., 2015). Recently, disease-associated microglia (DAM) have attracted the attention of scientists. DAM are phagocytic cells conserved in mice and human and are associated with neurodegenerative diseases, such as AD and amyotrophic lateral sclerosis (Keren-Shaul et al., 2017). They are localized near amyloid plaques (Aβ) and participate in the dismantling and digestion of Aβ. Similar phagocytosis of DAM and macrophages suggests that there may also be a link between LXs and DAM. However, to date, we have not found studies on the roles of SPMs in DAM. The issue may become a hot spot of research 1 day in the future.

### 4.4 Astrocytes

In the CNS, as crucial players in maintaining brain homeostasis, astrocytes contribute to the formation of the BBB, secrete neurotrophic factors and modulate synaptic transmission. Although not all astrocytic responses attenuate inflammation, their predominant function is to protect the brain from injury by regulating the neuroinflammatory response (Cekanaviciute and Buckwalter, 2016). Anti-inflammatory and antioxidant effects of LXs have been characterized in astrocytes. LXA_4_ could inhibit IL-1β-induced IL-8 and intercellular cell adhesion molecule-1 (ICAM-1) expression in 1321N1 human astrocytoma cells, thus reducing the infiltration of immune cells (Decker et al., 2009). In neonatal rat astrocyte primary cultures suffering oxygen-glucose deprivation/recovery, LXA_4_ inhibited LTC4 and LTA4 biosynthesis as well as 5-LOX translocation through an extracellular signal-regulated kinase (ERK) signal transduction pathway (Wu et al., 2012a). In response to LPS-induced neurotoxicity, ATL suppressed the production of nitric oxide and PGE_2_ through an NF-κB-dependent mechanism (Yao et al., 2014). In addition, LXA_4_ was found to downregulate the expression of aquaporin 4, which may be another anti-inflammatory target of LXA_4_ (Wu et al., 2019a). Furthermore, LXA_4_ could induce heme oxygenase-1 (HO-1) expression and glutathione (GSH) release as well as Nrf2 expression, of which nuclear translocation was partly ascribed to excess p62 accumulation, hence diminishing oxidative stress (Wu et al., 2015).

In the context of CNS inflammation, there is sophisticated crosstalk between astrocytes and other cells in the CNS (Linnerbauer et al., 2020). Moreover, the roles of astrocytes can be multifaceted. The regulation of astrocytes by LXs remains to be further studied. Whether LXs can regulate the communication between astrocytes and other cells cannot be ignored, either.

In addition to the cells mentioned above, LXs also regulate the activation and migration of leukocytes (Liu et al., 2019), exert anti-inflammatory and antiangiogenic effects on endothelial cells (Baker et al., 2009), and modulate activated endothelial leukocyte interactions (Smith et al., 2015), which are not discussed here.

## 5 Protective Effects of Lipoxins Against Neurological Diseases

### 5.1 Ischemic Stroke

The exact mechanisms responsible for ischemic stroke are not fully understood. Inflammation following ischemia-reperfusion plays a pivotal role in the pathophysiology of ischemic stroke and related brain injury (Mizuma and Yenari, 2017; Rajkovic et al., 2018). The generation of LXA_4_ after ischemic stroke has been detected in both animal models and clinical patients. Marcheselli et al. (2003) determined the LXA_4_ production in the hippocampus of mice after 1 h of middle cerebral artery occlusion (MCAO) followed by reperfusion and found a tendency for an increase in plasma LXA_4_ levels after injury, which peaked within 8 h and lasted for 24 h. Similarly, plasma LXA_4_ levels were measured, and it was determined that they increase in rats after global cerebral ischemia (GCI). Due to the long interval between observations, LXA_4_ did not change up to 6 h but tended to increase at 24 and 72 h and remained elevated until 168 h post-GCI (Jung et al., 2020). In the blood of patients after ischemic stroke, there was also a significant increase in LXA_4_ on the seventh day after the incident, indicating that LXA_4_ is one of the most important derivatives after an early incident of ischemic stroke (Szczuko et al., 2020).

The neuroprotection of LXs has been well established in ischemic stroke. It was first evaluated by Sobrado et al. (2009), who demonstrated that intracerebroventricular administration of LXA_4_ (1 nmol) caused a decrease in both infarct volume and neurological deficit scores after MCAO and confirmed that it was partially mediated by PPARγ. Then, You Shang et al. conducted further investigation on the efficacy of LXs using LXA_4_ ME in the same model and reconfirmed the neuroprotection of LXs in ischemic stroke (Wu Y. et al. 2010). They found that LXA_4_ ME could suppress neutrophil infiltration and lipid peroxidation levels, inhibit the activation of microglia and astrocytes and modulate the ratio of proinflammatory cytokines and anti-inflammatory cytokines, which were associated with the inhibition of the NF-κΒ pathway (Wu et al., 2010; Ye et al., 2010). In a later experiment, they demonstrated that LXA_4_ ME could also improve blood-brain barrier (BBB) integrity through the upregulation of metallopeptidase inhibitor-1 and the subsequent downregulation of matrix metallopeptidase (MMP)-9 expression and activity (Wu et al., 2012b). Recently, it was also shown that LXA_4_ exerted a neuroprotective effect on ischemic stroke by regulating microglial M1/M2 polarization *via* the Notch signaling pathway (Li et al., 2021). In accordance with these findings, it has been shown that BML-111, another LXA_4_ analog, could also alleviate neuroinflammation and maintain BBB integrity after ischemic stroke by decreasing the levels of MMP-9 and MMP-3 and protecting tight junction proteins in an ALX-dependent manner (Hawkins et al., 2014). In the following experiment, researchers attempted to further confirm the long-term effects of BML-111 on neurological recovery at 4 weeks after ischemic stroke in rats, but frustratingly, they failed (Hawkins et al., 2017). Vital et al. have focused on the ATL effects within the cerebral microvasculature. They found that ATL activated FPR2/3 and inhibited leukocyte-endothelial interactions to initiate endogenous pro-resolution (Smith et al., 2015) and inhibited neutrophil-platelet aggregation to prevent secondary embolism (Vital et al., 2016).

Le Wu et al. explored the anti-inflammatory and antioxidant mechanisms underlying the neuroprotective effects of LXA_4_ in ischemic stroke. They demonstrated that LXA_4_ could inhibit 5-LOX translocation and leukotriene biosynthesis both *in vivo* and *in vitro*, which are partly mediated by FPR2/ALX and through an ERK signal transduction pathway (Wu et al., 2012a). They also confirmed *in vivo* and *in vitro* that LXA_4_ could induce Nrf2 expression and its nuclear translocation, as well as HO-1 expression and GSH synthesis (Wu et al., 2013; Wu et al., 2015). Of interest, these pathways may be independent of FPR2/ALX and more closely related to p62 accumulation.

Cognitive impairment and depression are the most common complications of stroke, and it seems that LXs are associated with the outcomes. In terms of poststroke cognitive impairment (PSCI), it was reported that, compared with patients without PSCI, the levels of LXA_4_ were significantly reduced in PSCI patients, and the LXA_4_ levels were positively correlated with the Mini-Mental State Examination scores (Wang et al., 2021). Inspiringly, LXA_4_ pretreatment has been verified to improve cognitive function in aged rats after global cerebral ischemia-reperfusion (Wu et al., 2018). Regarding poststroke depression, it was shown that the changes in the Beck Depression Inventory-II scores of patients after stroke were inversely correlated with LXA_4_ level, hinting that LXA_4_ may also be a protective factor for the prevention of depression after stroke (Kotlega et al., 2020). In addition, it was investigated that the activation of FPR 2/3 could inhibit the action of glucocorticoids on the hypothalamic-pituitary-adrenal axis and maintain hippocampal homeostasis by preventing neuronal damage associated with depression (Peritore et al., 2020). Therefore, it is conceivable that LXs may confer neuroprotection in depression through the activation of FPR 2/3.

For ischemic stroke, diabetes mellitus is one of the major risk factors, and atherogenesis is the most common etiology. Excitingly, LXA_4_ has been reported to protect against inflammatory reactions in diabetic cerebral ischemia/reperfusion (I/R) injury, and its mechanism may be related to the inhibition of TNF-α and NF-κB expression (Han et al., 2016). Besides, LXA_4_ and ATL could compromise foam cell formation, oxidized low-density lipoprotein-induced inflammation and apoptotic signaling in macrophages during atherogenesis, which could be instructive for ischemic stroke prevention (Petri et al., 2017; Mai et al., 2018).

In conclusion, LXs are a promising therapeutic agent to better resolve the aggressive inflammatory state after ischemic stroke and limit irrecoverable neuronal damage. At present, the observed neuroprotective effects of LXA_4_ can last at least 72 h in the model of MCAO/reperfusion when administered immediately after ischemia (Wu et al., 2012a). Although BML-111 failed to show long-term neuroprotective effects, it remains to be explored whether other LX analogs can be protective up to weeks following ischemia and contribute to long-term functional recovery. On the other hand, in terms of LX administration, which dose to use, when to administer it, and how frequently may influence the results, offering new ideas for us to obtain a long-term protection from LXs (Han et al., 2016).

### 5.2 Hemorrhagic Stroke

Subarachnoid hemorrhage and intracerebral hemorrhage (ICH) are the two types of hemorrhagic stroke, in which inflammation is a vital pathologic manifestation of early brain injury and a crucial factor related to the outcome (Lucke-Wold et al., 2016; Xue and Yong, 2020). It has been confirmed that LXs can exert protective effects in SAH. The expression of endogenous LXA_4_ was decreased and its receptor FPR2/ALX was increased in the hippocampal and cerebral arteries after SAH, indicating a depletion of anti-inflammation (Guo et al., 2016). Exogenously injected LXA_4_ (1.0 nmol) at 1.5 h after SAH could significantly alleviate the pathology of SAH, including reducing brain edema, preserving BBB integrity, improving neurological scores, and enhancing spatial learning and memory abilities. In this experiment, the FPR2/p38 MAPK signaling pathway was shown to be involved in the anti-inflammatory pathway elicited by LXA_4_ (Guo et al., 2016). In addition, LXA_4_ could significantly ameliorate endothelial dysfunction, recover microflow and protect against inflammation by inhibiting neutrophil infiltration. The beneficial effects of LXA_4_ on endothelial function might be partly dependent on the inhibition of NF-κB via the FPR2/ERK1/2 pathway (Liu et al., 2019).

The preemptive treatment of unruptured intracranial aneurysms (IAs) is the first goal in the prevention of SAH. Frustratingly, except for open surgery and endovascular intervention, there is no noninvasive medical treatment for IAs. By targeting inflammation, nonsteroid anti-inflammatory drugs (NSAIDs) and statins exert a suppressive effect on IAs (Aoki et al., 2008; Hasan et al., 2011). Recently, eicosapentaenoic acid, the precursor of resolvin E1 targeting GPR120, was also reported to suppress the size of IAs and degenerative changes in the media in rats by suppressing NF-κB activation (Abekura et al., 2020). Considering the strong modulation of inflammation of SPMs, LXs may also have an effect on IAs. LXA_4_ may serve as an alternative treatment for SAH and is worthy of further exploration.

For ICH, it has been reported that LXA_4_ ME could inhibit neuronal apoptosis, decrease the levels of proinflammatory cytokines and improve neurologic function by inhibiting the NF-kB-dependent MMP-9 pathway in a rat model of ICH (Song et al., 2019). LX treatment may be a potential therapy after brain hemorrhage. In the future, more studies are needed to determine the potential role of LXs in ICH and to clarify the protective mechanism.

### 5.3 Neonatal Hypoxia-Ischemia Encephalopathy

Neonatal hypoxia-ischemia (HI) encephalopathy is the most common clinical brain injury in the perinatal period (Hagberg et al., 2015). Recently, the neuroprotective effects of LXA_4_ were reported in a rat model of neonatal HI brain injury (Zhu et al., 2020). LXA_4_ treatment suppressed acute inflammation and oxidative stress following brain injury in addition to preventing BBB disruption by regulating the IκB/NF-κB signaling pathway, which consequently attenuated HI brain damage. Furthermore, LXA_4_ succeeded in exerting long-term neuroprotection, which involved promoting the recovery of neuronal function and tissue structure 7 days post-HI and ameliorating motor and learning functioning 21 days after HI. Moreover, LXA_4_ significantly inhibited neuronal apoptosis both *in vivo* and *in vitro* experiments. Although there is a lack of sufficient research support, LXA_4_ raises new hope for HI, and further studies are required to elucidate the mechanisms underlying protection. Additionally, it is valuable to explore whether combined LXA_4_ treatment with hypothermia, the only effective treatment recognized in HI clinical practice, can produce more pronounced improvements in HIE.

### 5.4 Traumatic Brain Injury

In traumatic brain injury (TBI), following mechanical damage from an impact, called “primary injury,” a complex cascade of physiologic reactions will result in a secondary injury, among which primary BBB disruption and inflammatory response are the critical pathological steps (Jassam et al., 2017). AA products involving 5-HETE, 12-HETE and TXB2 were all increased in patients’ CSF 1–4 days following TBI (Shearer and Walker, 2018). In contrast, plasma LXA_4_ levels showed a tendency to decrease after TBI from 6 h up to 168 h in a controlled cortical impact rat model of TBI (Jung et al., 2020). These results indicate that proinflammatory activity rather than resolving activity is the dominant theme in TBI, which may lead to detrimental consequences, including increased intracranial pressure, brain edema, and brain herniation.

Neuroinflammation is proposed as an important manipulable aspect of secondary injury in animal and human studies. LXA_4_ treatment was shown to effectively reduce BBB permeability, brain edema and lesion volume 24 h post-TBI in mice (Luo et al., 2013). These protective effects of LXA_4_ were associated with the inhibition of proinflammatory cytokines (TNF-α, IL-1β and IL-6) and the activation of MAPK pathways (p-ERK and p-JNK but not p-p38). Of interest, LXA_4_ enhanced the activation of ALXR in astrocytes instead of microglia at 24 h after injury, but the exact mechanism is still unclear. The limitation of the study was that it only focused on the effects of LXA_4_ on the early changes within 24 h after TBI. In fact, the efficacy of TBI therapies is influenced by the type (focal vs. diffuse), stage (acute vs. chronic), severity, and location of the injury. Moreover, TBI can cause lifelong and dynamic influences such as sustained cognitive and psychiatric disorders, sleep-wake disturbances and neurodegeneration (Moretti et al., 2012; Sandsmark et al., 2017; Wilson et al., 2017). Therefore, more studies about the protective effect of LXs on TBI remain to be conducted.

### 5.5 Spinal Cord Injury

After spinal cord injury (SCI), incomplete or delayed resolution usually occurs and can lead to detrimental effects, including propagated tissue damage and impaired wound healing. First, the clearance of inflammatory cells containing neutrophils, macrophages, microglia and lymphocytes was impaired after SCI. Second, the synthesis of SPMs was delayed after contusion injury. The levels of 12-HETE and 15-HETE, which are pathway markers of the synthesis of LXA_4_, did not increase until 14 days after injury (Francos-Quijorna et al., 2017).

Exogenous administration of LXs has shown protective effects against SCI in animal models. LXA_4_ suppressed the damage induced by I/R in rabbits through its antiapoptotic and antioxidant activities (Liu et al., 2015). In a model of spinal cord hemisection, LXA_4_ inhibited microglial activation, moderated neuroinflammation and attenuated mechanical allodynia (Martini et al., 2016). Moreover, LXA_4_ upregulated Akt/Nrf2/HO-1 signaling, contributing to the improvement in functional recovery, attenuation of allodynia and hyperalgesia, reduction of lesion volume and inhibition of apoptotic signaling after SCI (Lu et al., 2018). Recently, BML-111 has also been reported to protect against SCI by suppressing inflammation and oxidative stress (Liu J. et al., 2020). Collectively, LXs may be considered a potential therapeutic agent for SCI and its associated complications.

### 5.6 Alzheimer’s Disease

The AD brain is marked by the accumulation of extracellular senile plaques and intracellular neurofibrillary tangles composed of Aβ and hyperphosphorylated-tau protein (p-tau), respectively. The etiological mechanisms underlying these neuropathological changes remain unclear, but dysregulation of glial cells, especially microglia, and elevated neuroinflammation make great contributions to disease progression (Heneka et al., 2015). The decreased levels of LXA_4_ are in line with the fact that the inflammation fails to be resolved in the AD brain. In the brain of 3xTg-AD mice, the levels of LXA_4_ were significantly lower than those in the brains of nontransgenic mice. As the best-known risk factor for AD, age reduced LXA_4_ levels with a pattern that showed a greater impact in AD mice (Dunn et al., 2015). Similar results were also found in the hippocampus of 5xFAD mice (Kantarci et al., 2018) and neurons from APP/PS1 mice (Lee et al., 2018). The latter study also uncovered that the decrease in 15-R-LXA_4_ might be related to the reduction in neural sphingosine kinase 1. Generally, sphingosine kinase 1 can acetylate COX2 to increase SPM secretion, increase microglial phagocytosis and improve AD-like brain pathology, including abnormal inflammation and neuronal dysfunction.

Marianne Schultzberg’s team analyzed postmortem brain tissues and CSF samples from AD patients concerning the production of SPM. In accordance with the findings in mice, the levels of LXA_4_ were reduced in both the postmortem CSF and hippocampus of AD patients (Wang et al., 2015). The decline in LXA_4_ synthesis was independent of the enzyme 15-LOX-2 since its expression in AD brains was elevated. They also found a positive correlation between the Mini-Mental State Examination scores and the levels of LXA_4_, showing the importance of LXA_4_ in maintaining normal cognition. Of interest, they verified the AD-related alterations in the entorhinal cortex, but no difference was found with regard to LXA_4_, revealing its tissue-specific expression (Dunn et al., 2015). In the study, they also detected the level of FPR2/ALX and found an increase in AD brains, which would make the tissue more responsive to pro-resolving signaling. However, the influence of LXA_4_ on the chemotaxis and production of reactive oxygen species in phagocytic cells also occurs *via* FPR2/ALX (Tiffany et al., 2001), which makes the role of the FPR2/ALX in AD progression more complicated.

In AD, several lines of evidence have shown the neuroprotective effect of LXA_4_. It was first demonstrated in the cortex and hippocampus of mice and BV2 microglial cells exposed to Aβ1–42. LXA_4_ inhibited the production of IL-1β and TNF-α *via* the NF-κB signaling pathway both *in vivo* and *in vitro* (Wu et al., 2011). LXA_4_ also displayed neuroprotection against spatial memory impairment induced by Aβ1–40 in a cannabinoid 1 receptor-dependent manner in mice (Pamplona et al., 2012). Whether PPAR-γ mediates the neuroprotective effects of LXA_4_ remains unknown, but the levels of PPAR-γ were markedly higher in AD than in the control compensatory reaction to the decreased levels of LXA_4_ (Wang et al., 2015). In addition, LXA_4_ alleviated oxidative stress-driven neuroinflammation in rats by targeting redox-sensitive proteins, including heat shock protein 72 and HO-1 (Trovato et al., 2016).

ATL also exerted neuroprotective effects on AD-like pathology in mice. ATL switched microglia from the classic phenotype to the alternative phenotype, thus improving the phagocytic function of microglia. Altered microglia promoted clearance of Aβ deposits and ultimately reduced synaptotoxicity and restored cognitive function in Tg2576 mice. According to the study, ATL can activate FPR2/ALX and reduce NF-κB activation in astrocytes, sequentially potentiating the action of alternative microglia (Medeiros et al., 2013). However, there was no significant effect on Aβ_42_ phagocytosis in CHME-3 microglia using LXA_4_ (Zhu et al., 2016). Apart from reducing Aβ levels, ATL could also decrease the levels of p-tau and enhance the cognitive performance of 3xTg-AD mice (Dunn et al., 2015). The decrease in p-tau was in part due to the inhibition of tau kinases GSK-3β and p38 MAPK. Additionally, microglial and astrocyte reactivity was inhibited by ATL treatment.

Combining LXA_4_ with other SPMs is a promising strategy to reverse the neuroinflammatory process associated with AD pathology since different SPMs have distinct selective functions and can regulate the process at multiple levels. Strong support is provided by the fact that combined treatment with LXA_4_ and resolvin E1 resolved AD-associated neuroinflammation and restored cognitive deficits more effectively than LXA_4_ treatment alone in 5xFAD mice (Kantarci et al., 2018). There have been several investigations on AD patients treated with dietary supplementation with SPM precursors (Kotani et al., 2006; Janssen and Kiliaan, 2014). For instance, it was shown that dietary supplementation with 240 mg/d AA and docosahexaenoic acid for 90 days improved the immediate memory and attention scores of patients with mild cognitive dysfunction. However, clinical trials in which human AD patients are directly treated with SPMs to correct the deficiency have not yet been performed.

Previous studies on AD tended to focus on “anti-inflammation” rather than “pro-resolution.” Although epidemiological studies have suggested that patients with protracted NSAID use have a lower prevalence of dementia (McGeer et al., 1996), clinical trials with NSAIDs have thus far yielded disappointing results (Cunningham and Skelly, 2012; Cudaback et al., 2014; Heneka et al., 2015). In contrast to “anti-inflammatory” treatments, SPMs do not block the enzymatic activity to regulate the inflammatory process. Instead, they interact with specific receptors to promote the beneficial and restorative aspects of inflammation. The LX dysregulation in neurodegeneration is attracting more and more attentions (Kim et al., 2020). We think that the intervention of LXs based on inflammatory resolution may give AD a chance to return to normal.

### 5.7 Multiple Sclerosis

Excessive neuroinflammation is a crucial pathological hallmark of multiple sclerosis (MS). Gijs Kooij and his colleagues revealed that the majority of SPMs, including LXA_4_ and LXB_4_, were significantly reduced in MS and correlated with disease progression through targeted lipid metabololipidomics in the plasma of MS patients (Kooij et al., 2020). They also found that the expression of SPM-related biosynthetic enzymes and receptors in blood-derived leukocytes of MS patients was impaired. *In vitro*, LXA_4_ and LXB_4_ were found to reduce MS-derived monocyte activation and cytokine production, improve BBB function and inhibit monocyte transmigration in MS patients (Kooij et al., 2020). *In vivo*, LXA_4_ administration ameliorated clinical signs of experimental autoimmune encephalomyelitis (EAE) in mice by normalizing the EAE-induced spinal cord lipidome and modulating Th1 and Th17 responses (Derada Troletti et al., 2021), which further highlighted the potential clinical application of LXs for MS.

### 5.8 Chronic Cerebral Hypoperfusion

Chronic cerebral hypoperfusion (CCH) is a chronic and silent disease characterized by sustained defects in brain perfusion. In recent years, as increasing evidence suggests the critical roles of CCH in the initiation and progression of vascular dementia and AD (Duncombe et al., 2017), CCH has garnered increasing attention from scientists and clinicians. Interestingly, LXs have been shown to exert beneficial effects against cognitive and neuropathological changes in CCH. LXA_4_ ME could exert neuroprotection in CCH by regulating endoplasmic reticulum stress and macroautophagy (Jia et al., 2015). LXA_4_ ME could also attenuate oxidative injury and reduce neuronal apoptosis related to CCH through activation of the ERK/Nrf2 signaling pathway in the rat hippocampus (Jin et al., 2014). Therefore, it is conceivable that the application of LXs in the future might provide beneficial effects for patients with CCH and ameliorate the related decline in cognition.

### 5.9 Neuropathic Pain

Neuropathic pain is attributed to lesions affecting the somatosensory nervous system that alter its structure and function so that pain occurs spontaneously and responses to noxious and innocuous stimuli are pathologically amplified (Costigan et al., 2009). There is abundant evidence for the involvement of inflammatory mediators in neuropathic pain. The development of allodynia and hyperalgesia is correlated with the release of TNF-α, IL-1β and IL-6, whereas antagonism or their knockout leads to the opposite effect. The mechanism of their action is not well established; nevertheless, they can directly excite nociceptors or indirectly maintain sensory abnormalities by modulating synaptic transmission and pain hypersensitivity (Marchand et al., 2005). It has been determined that the spinal cord, especially the astrocytes within it, can express FPR2/ALX *in vivo* and in culture (Svensson et al., 2007; Abdelmoaty et al., 2013; Wang et al., 2014). LX treatment has been reported to alleviate neuropathic pain under various pathological conditions, including opioid-induced hyperalgesia, peripheral nerve injury and spinal cord injury. The effect of LX on analgesics is primarily associated with its anti-inflammatory and pro-resolution properties.

LXA_4_ ME attenuated morphine antinociceptive tolerance and withdrawal-induced hyperalgesia. This prevention was correlated with the inactivation of NF-κB, inhibition of proinflammatory cytokines (IL-1β, IL-6, and TNF-α), and upregulation of anti-inflammatory cytokines (IL-10 and transforming growth factor-β1). According to the authors’ perspective, the actions of LXA_4_ ME were achieved by interacting with the Toll-like receptor 4 cascade, which has been verified as a contributor to painful neuropathy (Tanga et al., 2005), rather than opioid receptors (Jin et al., 2012). However, another study revealed the involvement of the μ-receptor/phosphoinositide-3-kinase (PI3K)-Akt signaling/NAcht leucine-rich-repeat protein 1 (NALP1) inflammasome cascade in this process and found that ATL could block this signaling cascade (Tian et al., 2015).

In chronic constriction injury (CCI)-induced neuropathic pain, ALT potently suppresses thermal and mechanical hyperalgesia and significantly inhibits NALP1 inflammasome activation, caspase-1 cleavage, and IL-1β maturation (Li et al., 2013). Further investigation indicated that the analgesic effect of ATL was mediated by inhibiting spinal JAK2/STAT3 signaling through FPR2/ALX and hence suppressing spinal neuroinflammation in CCI rats (Wang et al., 2014). In line with this discovery, LXA_4_ exerted similar antinociceptive effects in a model of chronic dorsal root ganglia compression in rats (Sun et al., 2012). Moreover, LXA_4_ potently alleviated radicular pain in a rat model of noncompressive lumbar disc herniation by attenuating the activation of NF-κB/p65, p-ERK and p-JNK, but not p-p38, in the dorsal root ganglion (Miao et al., 2015). Another study showed that LXA_4_ inhibited NLRP3 activation and autophagy in the dorsal root ganglion by regulating the JNK1/beclin-1/PI3KC3 axis, thus participating in its analgesic effects (Jin et al., 2020).

LXA_4_ also exhibited analgesic activity against SCI-induced neuropathic pain, as evidenced by an increase in the mechanical paw withdrawal threshold in a model of SCI in both mice and rats (Martini et al., 2016; Lu et al., 2018). The antinociceptive effects of LXA_4_ were mediated by FPR2/ALX and might be partly attributable to the decline in TNF-α from microglia (Martini et al., 2016). In addition, LXs could prevent the phosphorylation of ERK and JNK in astrocytes by activating FPR2/ALX (Svensson et al., 2007; Hu et al., 2012).

Recently, roles of SPMs in neuropathic pain were elucidated, suggesting that SPM can be promising targets to counteract neuropathic pain (Leuti et al., 2021). SPM represent the endogenous inhibitors of transient receptor potential cation channel subfamily V member 1 (TRPV1), which has been confirmed with Resolvin E1 and protectin 1. To date, it remains unknown whether LXs has an effect on TRPV1. Due to the intricated relationship between inflammation and pain, it is necessary to better understand the cellular and molecular pathways associated to the analgesia of LXs.

### 5.10 Others

In recent years, the protection of LXs has been underscored in a variety of central nervous system infections. First, LXs generated in a 5-LO-dependent manner have been demonstrated to control proinflammatory and type 1 T helper cells’ immune responses against *M. tuberculosis* infection (Bafica et al., 2005). Aspirin can improve outcomes from tuberculosis meningitis, which is speculated to be related to the production of ATL (Tobin et al., 2012). However, in a randomized placebo-controlled trial of adjunctive aspirin treatment in adults with tuberculosis meningitis, ATL concentrations were not changed by aspirin treatment, but it confirmed that SPM concentrations, including ATL in CSF, were associated with disease severity and outcome (Colas et al., 2019). In *Plasmodium berghei*-infected mice, LXA_4_ not only prolonged survival by inhibiting IL-12 production and CD8(+) IFN-γ(+) T cells (Shryock et al., 2013) but also ameliorated endothelial dysfunction by modulating ICAM-1 and HO-1 expression in brain tissue (Souza et al., 2015). Moreover, by generating LXA_4_ (Aliberti et al., 2002), wild-type mice avoid succumbing to encephalitis after *Toxoplasma gondii* infection. Notably, it has been proven that FPR2/ALX plays a pivotal role in glial cell activation in bacterial infection of the CNS (Braun et al., 2011), hinting that LXs may also have an effect on bacterial meningitis.

There is also evidence of the neuroprotective effect of LXs on epilepsy and retinal diseases. The LXA_4_ level and FPR2/ALX expression in the cortex and hippocampi of rats were greater in pentylenetetrazole-kindled rats than in the saline group. Aspirin can downregulate the levels of FPR2/ALX and LXA_4_, elevating the seizure threshold and helping achieve seizure control (Abd-Elghafour et al., 2017). Astrocyte-derived LXA_4_ and LXB_4_ have been reported to have a protective effect on the retina against acute kainic acid-induced injury and chronic glaucoma *in vivo* and to simultaneously dampen paraquat-induced oxidative stress *in vitro* (Livne-Bar et al., 2017). Recently, it was further characterized that intravitreal injection of LXA_4_ delayed the progression of retinal degeneration in mice through the modulation of microglial activities to suppress retinal inflammation and rescue photoreceptors (Lu et al., 2019).

## 6 Conclusion and Perspectives

LXs present opportunities to intervene in and promote human brain health. The neuroprotection of LXs has been well established in CNS cell types (Table 1). LXs can exert an array of protective effects on neurological diseases, including ischemic or hemorrhagic stroke, neonatal hypoxia-ischemia encephalopathy, brain and spinal cord injury, AD, MS, CCH, and neuropathic pain, showing great therapeutic potential for neuroinflammatory and neurodegenerative disorders (Table 2).

**TABLE 1:** Summary of *in vitro* studies on the neuroprotective effects of lipoxins.

Cell type	Model	Agent	Effects	References
Neural stem cells				
Murine neural stem cells	—	ATL, LXA_4_	Attenuated growth of NSCs by inducing the expression of epidermal growth factor receptor, cyclin E, p27, and caspase 8	Wada et al. (2006)
Neurons				
SH-SY5Y cells	STS-induced neurotoxicity	LXA_4_	Anti-apoptosis by targeting GPR32	Zhu et al. (2016)
HT-22 cells	Glutamate-induced neurotoxicity	LXA_4_, LXB_4_	LXA_4_: cell death reduced by targeting FPR2/ALX; LXB_4_: cell death reduced by influencing mitochondrial activity	Livne-Bar et al. (2017)
Rat primary cortical neurons	OGD	LXA_4_	Anti-apoptosis, anti-inflammation and anti-oxidation by inhibiting IκB/NF-κB pathway	Zhu et al. (2020)
Mouse primary cortical neurons	Serum deprivation	LXA_4_, LXB_4_	Cell death reduced only by LXB_4_	Livne-Bar et al. (2017)
RGCs	PQ-induced oxidative stress	LXA_4_, LXB_4_	LXA_4_: RGC survival rescued; LXB_4_: both RGC survival and neurite degeneration rescued	Livne-Bar et al. (2017)
Microglia				
BV2 cells	Stimulated by LPS	ATL	NO, iNOS, IL-1β and TNF-α reduced by inhibiting NF-κB, ERK, p38 MAPK and AP-1 signaling pathways; ROS reduced by inhibiting the function of NADPH oxidase; regulated the activation and polarization of microglia via the Notch Signaling Pathway	Wang et al. (2011), Wu et al. (2012c), Wu et al. (2019b)
BV2 cells	OGDR	LXA_4_	Regulated the polarization of microglia through the Notch signaling pathway	Li et al. (2021)
BV2 cells	Stimulated by Aβ_1-42_	LXA_4_	IL-1β and TNF-α reduced by inhibiting NF-κB signal pathway	Wu et al. (2011)
Human CHME3 cells	Stimulated by Aβ_42_	LXA_4_	No significant effect on microglial activation and phagocytosis	Zhu et al. (2016)
Astrocytes				
Rat primary astrocytes	OGDR	LXA_4_	LTB_4_, LTC_4_ and 5-LOX nuclear translocation reduced involving ALXR/ERK pathway; anti-oxidation by activating Nrf2 pathway and increasing the level of HO-1, GSH, and p62	Wu et al. (2012a), Wu et al. (2015)
Rat primary astrocytes	Stimulated by LPS	ATL, LXA_4_	NO, PGE2, iNOS and COX-2 reduced by inhibiting NF-κB signal pathway; down-regulate the expression of AQP4	Yao et al. (2014), Wu et al. (2019a)
1321N1 human astrocytoma cells	IL-1β-induced stimulation	LXA_4_	IL-8 and ICAM-1 reduced by inhibiting NF-κB signal pathway	Decker et al. (2009)

AP-1, activating protein-1; ATL, aspirin-triggered lipoxin A₄; Aβ, β-amyloid; COX-2, cyclooxygenase 2; ERK, extracellular signal-regulated kinase; FPR2/ALX, formyl peptide receptor 2/LXA_4_ receptor; GPR, G protein-coupled receptor; GSH, glutathione; HO-1, heme oxygenase; IFN, interferon; IL, interleukin; iNOS, inducible nitric oxide synthase; IκB, inhibitor κB; LOX, lipoxygenase; LPS, lipopolysaccharide; LT, leukotriene; LX, lipoxin; MAPK, mitogen-activated protein kinase; NADPH, nicotinamide adenine dinucleotide phosphate; NF-κB, nuclear factor kappa B; NO, nitric oxide; Nrf2, nuclear factor erythroid 2-related factor 2; OGD/R, oxygen-glucose deprivation/recovery; PQ, paraquat; RGCs, retinal ganglion cells; ROS, reactive oxygen species; STS, staurosporine; TNF, tumor necrosis factor.

**TABLE 2:** Summary of *in vivo* studies on the neuroprotective effects of lipoxins.

Disease type	Object	Substance	Outcome	Mechanism	References
Ischemia/reperfusion injury	Rat; mice	LXA_4_, LXA_4_ ME, ATL, BML-111	Infarct volume, brain water content, tissue damage, hemorrhagic transformation, neurologic deficit and cognitive impairment attenuated; BBB dysfunction ameliorated; the reactivity of the cerebral microvasculature inhibited; the cognitive function improved	Anti-apoptosis; inhibition of neutrophil infiltration, lipid peroxidation, and astrocyte activation; anti-inflammation; inhibition of 5-LOX translocation and leukotriene biosynthesis; downregulation of MMP-9 and MMP-3 expression and upregulation of TIMP-1 expression; involvement of the ERK signal transduction pathway; PPARγ agonistic actions; activation of neutrophil FPR2/3 regulating leukocyte-endothelial interactions and NPA formation; activation of Nrf2/HO-1/GSH signaling	Sobrado et al. (2009), Wu et al. (2010), Ye et al. (2010), Wu et al. (2012a), Wu et al. (2013), Hawkins et al. (2014), Smith et al. (2015), Han et al. (2016), Vital et al. (2016), Hawkins et al. (2017), Wu et al. (2018)
Intracerebral Hemorrhage	Rat	LXA_4_ ME	Neuronal apoptosis and cerebral edema reduced; neurologic function improved; the levels of proinflammatory cytokines decreased	Inhibition in NF-kB-dependent MMP-9 pathway	Song et al. (2019)
Subarachnoid hemorrhage	Rat	LXA_4_	Brain water content and BBB permeability decreased; neurological functions and spatial learning and memory abilities improved; cerebrovascular endothelial dysfunction ameliorated; microflow recovered	Anti-inflammation (FPR2/p38 MAPK pathway); suppression infiltration of neutrophils; inhibition of NF-κB via the FPR2/ERK1/2 pathway	Guo et al. (2016), Liu et al. (2019)
Hypoxia/ischemia neonatal brain injury	Rat	LXA_4_	Cerebral edema, infarct volume, and inflammatory responses reduced; neuronal function and tissue structure recovered; motor, learning and memory functions ameliorated; the integrity of the BBB maintained	Anti-inflammation; anti-apoptosis; anti-oxidation; inhibition of IκB/NF-κB pathway	Zhu et al. (2020)
Traumatic brain injury	Mice	LXA_4_	Cerebral edema, infarct volume and BBB breakdown reduced	Anti-inflammation; downregulation MAPK pathway with FPR2/ALX in astrocytes	Luo et al. (2013)
Spinal cord injury	Rabbit	LXA_4_	Neurological function improved; allodynia and hyperalgesia attenuated; lesion reduced	Anti-apoptosis; anti-oxidation; upregulation of Akt/Nrf2/HO-1 signaling	Liu et al. (2015), Martini et al. (2016), Lu et al. (2018), Liu J. et al. (2020)
Alzheimer’s disease	Mouse	ATL, BML-111	Cognitive impairment reduced; the expression of synaptic proteins increased; the levels of p-tau and Aβ reduced	Anti-inflammation; anti-oxidation; activation of microglia in a non-phlogistic phenotype; suppression of NF-κB activation; anti-apoptosis; modulation of CB1 receptors; inhibition of the tau kinases GSK-3β and p38 MAPK.	Wu et al. (2011), Pamplona et al. (2012), Medeiros et al. (2013), Dunn et al. (2015), Kantarci et al. (2018)
Multiple sclerosis	Mouse	LXA4	Clinical signs of experimental autoimmune encephalomyelitis ameliorated	Modulation of Th1 and Th17 response and the EAE-induced spinal cord lipidom	Derada Troletti et al. (2021)
Chronic cerebral hypoperfusion	Rat	LXA_4_ ME	Cognitive impairment reduced	Activation of ERK/Nrf2 signaling pathway; regulation of endoplasmic reticulum stress and macroautophagy	Jin et al. (2014), Jia et al. (2015)
Neuropathic pain	Mice; rat	LXA_4_, ATL, LXA_4_ ME	Mechanical allodynia in opioid-induced hyperalgesia, peripheral nerve injury and spinal cord injury attenuated	Anti-inflammation; inhibition of microglial activation through FPR2/ALX; inhibition of JAK2/STAT3 signaling; inactivation of NF-κB, ERK and p-JNK; inhibition of μ-receptor/PI3k-Akt signaling/NALP1 inflammasome cascade; anti- autophagy by regulating the JNK1/beclin-1/PI3KC3 axis	Tanga et al. (2005), Sun et al. (2012), Li et al. (2013), Wang et al. (2014), Miao et al. (2015), Tian et al. (2015), Martini et al. (2016), Lu et al. (2018), Jin et al. (2020)
Plasmodium berghei- infection	Mice	LXA_4_	Survival prolonged, endothelial dysfunction ameliorated	Inhibition of IL-12 production and CD8(+) IFN-γ (+) T cells; modulation of ICAM-1 and HO-1 expression	Shryock et al. (2013), Souza et al. (2015)
Toxoplasma gondii infection	Mice	LXA_4_	Survival prolonged	Regulation of proinflammatory responses	Aliberti et al. (2002)
Retinal diseases	Mice	LXA_4_, LXB_4_	The progression of retinal degeneration delayed; photoreceptors rescued	Modulation of microglial activities and anti-inflammation	Livne-Bar et al. (2017), Lu et al. (2019)

Akt, protein kinase B, PKB; ALXR, lipoxin A4 receptor; Aβ, Amyloid-beta; BBB, blood-brain barrier; CB1, cannabinoid receptor 1; ER, endoplasmic reticulum; ERK, extracellular signal-regulated kinase; FPR2/ALX, formyl peptide receptor 2/LXA_4_ receptor; GSH, glutathione; HO-1, heme oxygenase-1; IκB, inhibitor κB; JAK2, Janus kinase 2; JNK, c-Jun N-terminal kinase; LOX, lipoxygenase; LX, lipoxin; LXA_4_ ME, lipoxin A_4_ methyl ester; MAPK, mitogen-activated protein kinase; MMP, matrix metalloproteinase; NALP1, NAcht leucine-rich-repeat protein 1; NF-κB, nuclear factor kappa B; NPA, neutrophil-platelet aggregation; Nrf2, nuclear factor erythroid 2-related factor 2; PI3k, phosphoinositide-3-kinase; PPAR, peroxisome proliferator-activated receptor; SOCS, suppressors of cytokine signaling; STAT3, signal transducer and activator of transcription 3; TIMP, metallopeptidase inhibitor.

In terms of the treatment efficiency and potential risks, LXs might show superior advantages among the clinical therapeutic options for neurological diseases in the future. On the one hand, LXs present notable potency at a microgram dose of the compound and manifest protection in a broad spectrum of diseases not limited to the CNS, such as type 2 diabetes mellitus, hypertension, and coronary heart disease (Das, 2018). This means that applying LXs in patients with both neurological disorders and others diseases can benefit more than we expected. On the other hand, as an innate SPM, LXs are involved in physiological functions of the body and can be rapidly metabolized and inactivated, which may signify little interference with the healthy physiological process and few side effects for patients. Nevertheless, inflammation is a complex course. In acute events of the CNS, such as stroke, applying LXs at an incorrect time or in an overdose might impede the natural processes of focus clearance and functional recovery. In chronic neuroinflammation and neurodegeneration, whether persistence of anti-inflammation by LXs could impair innate immune responses and result in adverse effects, such as neoplasia, is an issue worth considering. In addition, improper use of LXs may possibly augment the effects of resolution. For instance, overdose of LXs might result in excessive phagocytosis of macrophages leading to synapse destruction. However, all above are based on our speculations. In the future, more studies on the adverse effects of LXs are essential before their clinical application.

Before the extensive use of LXs in humans, there are also other important issues for researchers to consider. Regarding pharmacodynamic, to date, most studies have interpreted the neuroprotective effects of LXs with their pro-resolving activity in neuroinflammation. Whether LXs exert direct protective effects in the CNS remains to be seen, and more research is needed to understand the specific role that LXs play in each given disease. Concerning pharmacokinetics, there are two major factors to consider. To our knowledge, no studies have provided explicit evidence for the BBB permeability of LXs. If LXs are beneficial in CNS diseases only when administered by intrathecal injection instead of oral or intravenous administration, they will cause great pain to patients due to repeated punctures and will be limited in use. The other major factor is that natural LXs are characterized by rapid metabolic inactivation, temperature sensitivity and a lack of tissue specificity. Chemical modification of LX structures and the development of more LX analogs could render them more effective for therapeutic use in nervous diseases. Recently, a drug delivery system composed of neutrophil membrane-derived nanovesicles was loaded with SPMs specifically targeting inflamed brain endothelium during I/R, thus protecting against brain damage during ischemic stroke (Dong et al., 2019). Advanced nanocarrier provides a strategy for lipoxin-related drug design. We believe that along with the advances in drug design, LXs will spring more new surprises down our road against neurological diseases.

## Glossary

AA: arachidonic acid
AD: Alzheimer’s disease
Akt: protein kinase B
ATL: aspirin-triggered lipoxins
ATLA_4_: aspirin-triggered lipoxin A_4_
ATLB_4_: aspirin-triggered lipoxin B_4_
Aβ: amyloid β
BBB: blood-brain barrier
CCH: chronic cerebral hypoperfusion
CNS: central nervous system
COX-2: cyclooxygenase-2
CSF: cerebrospinal fluid
DAM: disease-associated microglia
ERK: extracellular signal-regulated kinase
FPR2: formyl peptide receptor 2
GPR: G protein-coupled receptor
HI: hypoxia-ischemia
HO-1: heme oxygenase-1
IAs: intracranial aneurysms
ICAM-1: intercellular cell adhesion molecule-1
ICC: immunocytochemistry
ICH: intracerebral hemorrhage
IL: interleukin
JAK2: Janus kinase 2
LOX: lipoxygenase
LPS: lipopolysaccharide
LT: leukotriene
LXA_4_ ME: LXA_4_ methyl ester
LXs: lipoxins
MAP1LC3: microtubule-associated protein 1 light chain 3
MAPK: mitogen-activated protein kinase
MMP-9: matrix metallopeptidase 9
MS: multiple sclerosis
NADPH: abrogate nicotinamide adenine dinucleotide phosphate
NF-κB: nuclear factor kappa B
Nrf2: erythroid 2-related factor 2
NSAIDs: non-steroid anti-inflammatory drugs
NSCs: neural stem cells
PGE2: prostaglandin E2
PGs: prostaglandins
PI3k: phosphoinositide-3-kinase
PPARγ: peroxisome proliferator-activated receptors gamma
PSCI: post-stroke cognitive impairment
p-tau: phosphorylated-tau protein
ROS: reactive oxygen species
RT-PCR: reverse transcription-polymerase chain reaction
SAH: subarachnoid hemorrhage
SCI: spinal cord injury
SPMs: specialized pro-resolving mediators
STAT3: signal transducer and activator of transcription 3
TBI: traumatic brain injury
TNF: tumor necrosis factor
TXs: thromboxanes

## References

[B1] Abd-Elghafour B. A. El-Sayed N. M. Ahmed A. A. Zaitone S. A. Moustafa Y. M. (2017). Aspirin and (Or) omega-3 Polyunsaturated Fatty Acids Protect against Corticohippocampal Neurodegeneration and Downregulate Lipoxin A4 Production and Formyl Peptide Receptor-like 1 Expression in Pentylenetetrazole-Kindled Rats. Can. J. Physiol. Pharmacol. 95, 340–348. 10.1139/cjpp-2016-0060 28060522

[B2] Abdelmoaty S. Wigerblad G. Bas D. B. Codeluppi S. Fernandez-Zafra T. El-Awady S. (2013). Spinal Actions of Lipoxin A4 and 17(R)-resolvin D1 Attenuate Inflammation-Induced Mechanical Hypersensitivity and Spinal TNF Release. PLoS One 8 (9), e75543. 10.1371/journal.pone.0075543 24086560 PMC3782447

[B3] Abekura Y. Ono I. Kawashima A. Takizawa K. Koseki H. Miyata H. (2020). Eicosapentaenoic Acid Prevents the Progression of Intracranial Aneurysms in Rats. J. Neuroinflammation 17 (1), 129. 10.1186/s12974-020-01802-8 32331514 PMC7181479

[B4] Aliberti J. Serhan C. Sher A. (2002). Parasite-induced Lipoxin A4 Is an Endogenous Regulator of IL-12 Production and Immunopathology in Toxoplasma Gondii Infection. J. Exp. Med. 196 (9), 1253–1262. 10.1084/jem.20021183 12417634 PMC2194099

[B5] Aoki T. Kataoka H. Ishibashi R. Nozaki K. Hashimoto N. (2008). Simvastatin Suppresses the Progression of Experimentally Induced Cerebral Aneurysms in Rats. Stroke 39 (4), 1276–1285. 10.1161/STROKEAHA.107.503086 18309148

[B6] Ariel A. Chiang N. Arita M. Petasis N. A. Serhan C. N. (2003). Aspirin-triggered Lipoxin A4 and B4 Analogs Block Extracellular Signal-Regulated Kinase-dependent TNF-Alpha Secretion from Human T Cells. J. Immunol. 170 (12), 6266–6272. 10.4049/jimmunol.170.12.6266 12794159

[B7] Bafica A. Scanga C. A. Serhan C. Machado F. White S. Sher A. (2005). Host Control of *Mycobacterium tuberculosis* Is Regulated by 5-lipoxygenase-dependent Lipoxin Production. J. Clin. Invest. 115 (6), 1601–1606. 10.1172/JCI23949 15931391 PMC1136995

[B8] Baker N. O'Meara S. J. Scannell M. Maderna P. Godson C. Godson C. (2009). Lipoxin A4: Anti-inflammatory and Anti-angiogenic Impact on Endothelial Cells. J. Immunol. 182 (6), 3819–3826. 10.4049/jimmunol.0803175 19265161

[B9] Beaino W. Janssen B. Vugts D. J. de Vries H. E. Windhorst A. D. (2021). Towards PET Imaging of the Dynamic Phenotypes of Microglia. Clin. Exp. Immunol. 206, 282–300. 10.1111/cei.13649 34331705 PMC8561701

[B10] Becker E. L. Forouhar F. A. Grunnet M. L. Boulay F. Tardif M. Bormann B. J. (1998). Broad Immunocytochemical Localization of the Formylpeptide Receptor in Human Organs, Tissues, and Cells. Cell Tissue Res. 292 (1), 129–135. 10.1007/s004410051042 9506920

[B11] Birnbaum Y. Ye Y. Lin Y. Freeberg S. Y. Nishi S. P. Martinez J. D. (2006). Augmentation of Myocardial Production of 15-Epi-Lipoxin-A4 by Pioglitazone and Atorvastatin in the Rat. Circulation 114 (9), 929–935. 10.1161/CIRCULATIONAHA.106.629907 16908763

[B12] Braun B. J. Slowik A. Leib S. L. Lucius R. Varoga D. Wruck C. J. (2011). The Formyl Peptide Receptor Like-1 and Scavenger Receptor MARCO Are Involved in Glial Cell Activation in Bacterial Meningitis. J. Neuroinflammation 8 (1), 11. 10.1186/1742-2094-8-11 21299846 PMC3040686

[B13] Cai W. Yang T. Liu H. Han L. Zhang K. Hu X. (2018). Peroxisome Proliferator-Activated Receptor γ (PPARγ): A Master Gatekeeper in CNS Injury and Repair. Prog. Neurobiol. 163-164, 27–58. 10.1016/j.pneurobio.2017.10.002 29032144 PMC6037317

[B14] Cattaneo F. Parisi M. Ammendola R. (2013). Distinct Signaling Cascades Elicited by Different Formyl Peptide Receptor 2 (FPR2) Agonists. Int. J. Mol. Sci. 14 (4), 7193–7230. 10.3390/ijms14047193 23549262 PMC3645683

[B15] Cekanaviciute E. Buckwalter M. S. (2016). Astrocytes: Integrative Regulators of Neuroinflammation in Stroke and Other Neurological Diseases. Neurotherapeutics 13, 685–701. 10.1007/s13311-016-0477-8 27677607 PMC5081110

[B16] Clària J. Serhan C. N. (1995). Aspirin Triggers Previously Undescribed Bioactive Eicosanoids by Human Endothelial Cell-Leukocyte Interactions. Proc. Natl. Acad. Sci. U S A. 92 (21), 9475–9479. 10.1073/pnas.92.21.9475 7568157 PMC40824

[B17] Colas R. A. Nhat L. T. H. Thuong N. T. T. Gómez E. A. Ly L. Thanh H. H. (2019). Proresolving Mediator Profiles in Cerebrospinal Fluid Are Linked with Disease Severity and Outcome in Adults with Tuberculous Meningitis. FASEB J. 33 (11), 13028–13039. 10.1096/fj.201901590R 31500466 PMC6902685

[B18] Costigan M. Scholz J. Woolf C. J. (2009). Neuropathic Pain: a Maladaptive Response of the Nervous System to Damage. Annu. Rev. Neurosci. 32, 1–32. 10.1146/annurev.neuro.051508.135531 19400724 PMC2768555

[B19] Cudaback E. Jorstad N. L. Yang Y. Montine T. J. Keene C. D. (2014). Therapeutic Implications of the Prostaglandin Pathway in Alzheimer's Disease. Biochem. Pharmacol. 88 (4), 565–572. 10.1016/j.bcp.2013.12.014 24434190 PMC3972296

[B21] Cunningham C. Skelly D. T. (2012). Non-steroidal Anti-inflammatory Drugs and Cognitive Function: Are Prostaglandins at the Heart of Cognitive Impairment in Dementia and Delirium. J. Neuroimmune Pharmacol. 7 (1), 60–73. 10.1007/s11481-011-9312-5 21932048 PMC3280386

[B22] Das U. N. (2018). Ageing: Is There a Role for Arachidonic Acid and Other Bioactive Lipids? A Review. J. Adv. Res. 11, 67–79. 10.1016/j.jare.2018.02.004 30034877 PMC6052661

[B23] Decker Y. McBean G. Godson C. (2009). Lipoxin A4 Inhibits IL-1beta-induced IL-8 and ICAM-1 Expression in 1321N1 Human Astrocytoma Cells. Am. J. Physiol. Cel Physiol 296 (6), C1420–C1427. 10.1152/ajpcell.00380.2008 19357230

[B24] Derada Troletti C. Enzmann G. Chiurchiù V. Kamermans A. Tietz S. M. Norris P. C. (2021). Pro-resolving Lipid Mediator Lipoxin A4 Attenuates Neuro-Inflammation by Modulating T Cell Responses and Modifies the Spinal Cord Lipidome. Cell Rep 35, 109201. 10.1016/j.celrep.2021.109201 34077725 PMC8491454

[B25] Devanney N. A. Stewart A. N. Gensel J. C. (2020). Microglia and Macrophage Metabolism in CNS Injury and Disease: The Role of Immunometabolism in Neurodegeneration and Neurotrauma. Exp. Neurol. 329, 113310. 10.1016/j.expneurol.2020.113310 32289316 PMC7237336

[B26] Dong X. Gao J. Zhang C. Y. Hayworth C. Frank M. Wang Z. (2019). Neutrophil Membrane-Derived Nanovesicles Alleviate Inflammation to Protect Mouse Brain Injury from Ischemic Stroke. ACS Nano 13 (2), 1272–1283. 10.1021/acsnano.8b06572 30673266 PMC6424134

[B27] Duncombe J. Kitamura A. Hase Y. Ihara M. Kalaria R. N. Horsburgh K. (2017). Chronic Cerebral Hypoperfusion: a Key Mechanism Leading to Vascular Cognitive Impairment and Dementia. Closing the Translational gap between Rodent Models and Human Vascular Cognitive Impairment and Dementia. Clin. Sci. (Lond) 131 (19), 2451–2468. 10.1042/CS20160727 28963120

[B28] Dunn H. C. Ager R. R. Baglietto-Vargas D. Cheng D. Kitazawa M. Cribbs D. H. (2015). Restoration of Lipoxin A4 Signaling Reduces Alzheimer's Disease-like Pathology in the 3xTg-AD Mouse Model. J. Alzheimers Dis. 43 (3), 893–903. 10.3233/JAD-141335 25125468 PMC4319708

[B29] Feng X. Valdearcos M. Uchida Y. Lutrin D. Maze M. Koliwad S. K. (2017). Microglia Mediate Postoperative Hippocampal Inflammation and Cognitive Decline in Mice. JCI Insight 2 (7), e91229. 10.1172/jci.insight.91229 28405620 PMC5374063

[B30] Fiore S. Maddox J. F. Perez H. D. Serhan C. N. (1994). Identification of a Human cDNA Encoding a Functional High Affinity Lipoxin A4 Receptor. J. Exp. Med. 180 (1), 253–260. 10.1084/jem.180.1.253 8006586 PMC2191537

[B31] Francos-Quijorna I. Santos-Nogueira E. Gronert K. Sullivan A. B. Kopp M. A. Brommer B. (2017). Maresin 1 Promotes Inflammatory Resolution, Neuroprotection, and Functional Neurological Recovery after Spinal Cord Injury. J. Neurosci. 37 (48), 11731–11743. 10.1523/JNEUROSCI.1395-17.2017 29109234 PMC5707767

[B32] Godson C. Mitchell S. Harvey K. Petasis N. A. Hogg N. Brady H. R. (2000). Cutting Edge: Lipoxins Rapidly Stimulate Nonphlogistic Phagocytosis of Apoptotic Neutrophils by Monocyte-Derived Macrophages. J. Immunol. 164 (164), 1663–1667. 10.4049/jimmunol.164.4.1663 10657608

[B33] Guo Z. Hu Q. Xu L. Guo Z. N. Ou Y. He Y. (2016). Lipoxin A4 Reduces Inflammation through Formyl Peptide Receptor 2/p38 MAPK Signaling Pathway in Subarachnoid Hemorrhage Rats. Stroke 47 (2), 490–497. 10.1161/STROKEAHA.115.011223 26732571 PMC4729632

[B34] Hagberg H. Mallard C. Ferriero D. M. Vannucci S. J. Levison S. W. Vexler Z. S. (2015). The Role of Inflammation in Perinatal Brain Injury. Nat. Rev. Neurol. 11 (4), 192–208. 10.1038/nrneurol.2015.13 25686754 PMC4664161

[B35] Han J. Q. Liu C. L. Wang Z. Y. Liu L. Cheng L. Fan Y. D. (2016). Anti-inflammatory Properties of Lipoxin A4 Protect against Diabetes Mellitus Complicated by Focal Cerebral Ischemia/reperfusion Injury. Neural Regen. Res. 11 (4), 636–640. 10.4103/1673-5374.180750 27212926 PMC4870922

[B36] Hanisch U. K. Kettenmann H. (2007). Microglia: Active Sensor and Versatile Effector Cells in the normal and Pathologic Brain. Nat. Neurosci. 10 (11), 1387–1394. 10.1038/nn1997 17965659

[B37] Hasan D. M. Mahaney K. B. Brown R. D. Jr Meissner I. Piepgras D. G. Huston J. (2011). Aspirin as a Promising Agent for Decreasing Incidence of Cerebral Aneurysm Rupture. Stroke 42 (11), 3156–3162. 10.1161/STROKEAHA.111.619411 21980208 PMC3432499

[B38] Hawkins K. E. DeMars K. M. Alexander J. C. de Leon L. G. Pacheco S. C. Graves C. (2017). Targeting Resolution of Neuroinflammation after Ischemic Stroke with a Lipoxin A4 Analog: Protective Mechanisms and Long-Term Effects on Neurological Recovery. Brain Behav. 7, e00688. 10.1002/brb3.688 28523230 PMC5434193

[B39] Hawkins K. E. DeMars K. M. Singh J. Yang C. Cho H. S. Frankowski J. C. (2014). Neurovascular protection by post-ischemic Intravenous Injections of the Lipoxin A4 Receptor Agonist, BML-111, in a Rat Model of Ischemic Stroke. J. Neurochem. 129 (1), 130–142. 10.1111/jnc.12607 24225006 PMC3967241

[B40] Heneka M. T. Carson M. J. El Khoury J. Landreth G. E. Brosseron F. Feinstein D. L. (2015). Neuroinflammation in Alzheimer's Disease. Lancet Neurol. 14 (4), 388–405. 10.1016/S1474-4422(15)70016-5 25792098 PMC5909703

[B41] Ho C. F. Ismail N. B. Koh J. K. Gunaseelan S. Low Y. H. Ng Y. K. (2018). Localisation of Formyl-Peptide Receptor 2 in the Rat Central Nervous System and its Role in Axonal and Dendritic Outgrowth. Neurochem. Res. 43 (8), 1587–1598. 10.1007/s11064-018-2573-0 29948727 PMC6061218

[B42] Hu S. Mao-Ying Q. L. Wang J. Wang Z. F. Mi W. L. Wang X. W. (2012). Lipoxins and Aspirin-Triggered Lipoxin Alleviate Bone Cancer Pain in Association with Suppressing Expression of Spinal Proinflammatory Cytokines. J. Neuroinflammation 9, 278. 10.1186/1742-2094-9-278 23268791 PMC3558391

[B43] Janssen C. I. Kiliaan A. J. (2014). Long-chain Polyunsaturated Fatty Acids (LCPUFA) from Genesis to Senescence: the Influence of LCPUFA on Neural Development, Aging, and Neurodegeneration. Prog. Lipid Res. 53, 1–17. 10.1016/j.plipres.2013.10.002 24334113

[B44] Jassam Y. N. Izzy S. Whalen M. McGavern D. B. El Khoury J. (2017). Neuroimmunology of Traumatic Brain Injury: Time for a Paradigm Shift. Neuron 95 (6), 1246–1265. 10.1016/j.neuron.2017.07.010 28910616 PMC5678753

[B45] Jia Y. Jin W. Xiao Y. Dong Y. Wang T. Fan M. (2015). Lipoxin A4 Methyl Ester Alleviates Vascular Cognition Impairment by Regulating the Expression of Proteins Related to Autophagy and ER Stress in the Rat hippocampus. Cell Mol Biol Lett. 20 (3), 475–487. 10.1515/cmble-2015-0027 26208392

[B46] Jin H. Li Y. H. Xu J. S. Guo G. Q. Chen D. L. Bo Y. (2012). Lipoxin A4 Analog Attenuates Morphine Antinociceptive Tolerance, Withdrawal-Induced Hyperalgesia, and Glial Reaction and Cytokine Expression in the Spinal Cord of Rat. Neuroscience 208, 1–10. 10.1016/j.neuroscience.2012.02.009 22366510

[B47] Jin J. Xie Y. Shi C. Ma J. Wang Y. Qiao L. (2020). Lipoxin A4 Inhibits NLRP3 Inflammasome Activation in Rats with Non-compressive Disc Herniation through the JNK1/Beclin-1/PI3KC3 Pathway. Front. Neurosci. 14, 799. 10.3389/fnins.2020.00799 33071721 PMC7539067

[B48] Jin W. Jia Y. Huang L. Wang T. Wang H. Dong Y. (2014). Lipoxin A4 Methyl Ester Ameliorates Cognitive Deficits Induced by Chronic Cerebral Hypoperfusion through Activating ERK/Nrf2 Signaling Pathway in Rats. Pharmacol. Biochem. Behav. 124, 145–152. 10.1016/j.pbb.2014.05.023 24909072

[B49] Jung J. S. Kho A. R. Lee S. H. Choi B. Y. Kang S. H. Koh J. Y. (2020). Changes in Plasma Lipoxin A4, Resolvins and CD59 Levels after Ischemic and Traumatic Brain Injuries in Rats. Korean J. Physiol. Pharmacol. 24 (2), 165–171. 10.4196/kjpp.2020.24.2.165 32140040 PMC7043996

[B50] Kantarci A. Aytan N. Palaska I. Stephens D. Crabtree L. Benincasa C. (2018). Combined Administration of Resolvin E1 and Lipoxin A4 Resolves Inflammation in a Murine Model of Alzheimer's Disease. Exp. Neurol. 300, 111–120. 10.1016/j.expneurol.2017.11.005 29126887

[B51] Ke Y. Zebda N. Oskolkova O. Afonyushkin T. Berdyshev E. Tian Y. (2017). Anti-Inflammatory Effects of OxPAPC Involve Endothelial Cell-Mediated Generation of LXA4. Circ. Res. 121 (3), 244–257. 10.1161/CIRCRESAHA.116.310308 28522438 PMC5886749

[B52] Keren-Shaul H. Spinrad A. Weiner A. Matcovitch-Natan O. Dvir-Szternfeld R. Ulland T. K. (2017). A Unique Microglia Type Associated with Restricting Development of Alzheimer's Disease. Cell 169, 1276–e17. 10.1016/j.cell.2017.05.018 28602351

[B53] Kim C. Livne-Bar I. Gronert K. Sivak J. M. (2020). Fair-Weather Friends: Evidence of Lipoxin Dysregulation in Neurodegeneration. Mol. Nutr. Food Res. 64, e1801076. 10.1002/mnfr.201801076 31797529 PMC8691368

[B54] Kim N. Lannan K. L. Thatcher T. H. Pollock S. J. Woeller C. F. Phipps R. P. (2018). Lipoxin B4 Enhances Human Memory B Cell Antibody Production via Upregulating Cyclooxygenase-2 Expression. J. Immunol. 201 (11), 3343–3351. 10.4049/jimmunol.1700503 30348736 PMC6246818

[B55] Klegeris A. McGeer P. L. (2003). Toxicity of Human Monocytic THP-1 Cells and Microglia toward SH-Sy5y Neuroblastoma Cells Is Reduced by Inhibitors of 5-lipoxygenase and its Activating Protein FLAP. J. Leukoc. Biol. 73 (3), 369–378. 10.1189/jlb.1002482 12629151

[B56] Kooij G. Troletti C. D. Leuti A. Norris P. C. Riley I. Albanese M. (2020). Specialized Pro-resolving Lipid Mediators Are Differentially Altered in Peripheral Blood of Patients with Multiple Sclerosis and Attenuate Monocyte and Blood-Brain Barrier Dysfunction. Haematologica 105, 2056–2070. 10.3324/haematol.2019.219519 31780628 PMC7395264

[B57] Kotani S. Sakaguchi E. Warashina S. Matsukawa N. Ishikura Y. Kiso Y. (2006). Dietary Supplementation of Arachidonic and Docosahexaenoic Acids Improves Cognitive Dysfunction. Neurosci. Res. 56 (2), 159–164. 10.1016/j.neures.2006.06.010 16905216

[B58] Kotlega D. Zembron-Lacny A. Golab-Janowska M. Nowacki P. Szczuko M. (2020). The Association of Free Fatty Acids and Eicosanoids with the Severity of Depressive Symptoms in Stroke Patients. Int. J. Mol. Sci. 21, 5220. 10.3390/ijms21155220 PMC7432477 32717948

[B59] Lawrence T. Willoughby D. A. Gilroy D. W. (2002). Anti-inflammatory Lipid Mediators and Insights into the Resolution of Inflammation. Nat. Rev. Immunol. 2 (10), 787–795. 10.1038/nri915 12360216

[B60] Lee J. Y. Han S. H. Park M. H. Baek B. Song I. S. Choi M. K. (2018). Neuronal SphK1 Acetylates COX2 and Contributes to Pathogenesis in a Model of Alzheimer's Disease. Nat. Commun. 9 (1), 1479. 10.1038/s41467-018-03674-2 29662056 PMC5902554

[B61] Lee S. Nakahira K. Dalli J. Siempos I. I. Norris P. C. Colas R. A. (2017). NLRP3 Inflammasome Deficiency Protects against Microbial Sepsis via Increased Lipoxin B4 Synthesis. Am. J. Respir. Crit. Care Med. 196 (6), 713–726. 10.1164/rccm.201604-0892OC 28245134 PMC5620673

[B62] Lefer A. M. Stahl G. L. Lefer D. J. Brezinski M. E. Nicolaou K. C. Veale C. A. (1988). Lipoxins A4 and B4: Comparison of Icosanoids Having Bronchoconstrictor and Vasodilator Actions but Lacking Platelet Aggregatory Activity. Proc. Natl. Acad. Sci. U S A. 85 (21), 8340–8344. 10.1073/pnas.85.21.8340 3186729 PMC282425

[B63] Leuti A. Fava M. Pellegrini N. Maccarrone M. (2021). Role of Specialized Pro-resolving Mediators in Neuropathic Pain. Front. Pharmacol. 12, 717993. 10.3389/fphar.2021.717993 34456731 PMC8385637

[B64] Li Q. Tian Y. Wang Z. F. Liu S. B. Mi W. L. Ma H. J. (2013). Involvement of the Spinal NALP1 Inflammasome in Neuropathic Pain and Aspirin-Triggered-15-Epi-Lipoxin A4 Induced Analgesia. Neuroscience 254, 230–240. 10.1016/j.neuroscience.2013.09.028 24076348

[B65] Li Q. Q. Ding D. H. Wang X. Y. Sun Y. Y. Wu J. (2021). Lipoxin A4 Regulates Microglial M1/M2 Polarization after Cerebral Ischemia-Reperfusion Injury via the Notch Signaling Pathway. Exp. Neurol. 339, 113645. 10.1016/j.expneurol.2021.113645 33600815

[B66] Linnerbauer M. Wheeler M. A. Quintana F. J. (2020). Astrocyte Crosstalk in CNS Inflammation. Neuron 108, 608–622. 10.1016/j.neuron.2020.08.012 32898475 PMC7704785

[B67] Liu G. J. Tao T. Wang H. Zhou Y. Gao X. Gao Y. Y. (2020). Functions of Resolvin D1-Alx/fpr2 Receptor Interaction in the Hemoglobin-Induced Microglial Inflammatory Response and Neuronal Injury. J. Neuroinflammation 17 (1), 239. 10.1186/s12974-020-01918-x 32795323 PMC7429751

[B68] Liu J. Peng L. Li J. (2020). The Lipoxin A4 Receptor Agonist BML-111 Alleviates Inflammatory Injury and Oxidative Stress in Spinal Cord Injury. Med. Sci. Monit. 26, e919883. 10.12659/MSM.919883 31971927 PMC6996263

[B69] Liu L. Zhang P. Zhang Z. Hu Q. He J. Liu H. (2019). LXA4 Ameliorates Cerebrovascular Endothelial Dysfunction by Reducing Acute Inflammation after Subarachnoid Hemorrhage in Rats. Neuroscience 408, 105–114. 10.1016/j.neuroscience.2019.03.038 30910642

[B70] Liu Z. Q. Zhang H. B. Wang J. Xia L. J. Zhang W. (2015). Lipoxin A4 Ameliorates Ischemia/reperfusion Induced Spinal Cord Injury in Rabbit Model. Int. J. Clin. Exp. Med. 8 (8), 12826–12833. 26550197 PMC4612882

[B71] Livne-Bar I. Wei J. Liu H. H. Alqawlaq S. Won G. J. Tuccitto A. (2017). Astrocyte-derived Lipoxins A4 and B4 Promote Neuroprotection from Acute and Chronic Injury. J. Clin. Invest. 127 (12), 4403–4414. 10.1172/JCI77398 29106385 PMC5707141

[B72] Lu T. Wu X. Wei N. Liu X. Zhou Y. Shang C. (2018). Lipoxin A4 Protects against Spinal Cord Injury via Regulating Akt/nuclear Factor (Erythroid-derived 2)-like 2/heme Oxygenase-1 Signaling. Biomed. Pharmacother. 97, 905–910. 10.1016/j.biopha.2017.10.092 29136768

[B73] Lu Z. Zhang H. Zhang X. Gao Y. Yin Z. Q. (2019). Lipoxin A4 Delays the Progression of Retinal Degeneration via the Inhibition of Microglial Overactivation. Biochem. Biophys. Res. Commun. 516 (3), 900–906. 10.1016/j.bbrc.2019.06.137 31272712

[B74] Lucke-Wold B. P. Logsdon A. F. Manoranjan B. Turner R. C. McConnell E. Vates G. E. (2016). Aneurysmal Subarachnoid Hemorrhage and Neuroinflammation: A Comprehensive Review. Int. J. Mol. Sci. 17 (4), 497. 10.3390/ijms17040497 27049383 PMC4848953

[B75] Luo C. L. Li Q. Q. Chen X. P. Zhang X. M. Li L. L. Li B. X. (2013). Lipoxin A4 Attenuates Brain Damage and Downregulates the Production of Pro-inflammatory Cytokines and Phosphorylated Mitogen-Activated Protein Kinases in a Mouse Model of Traumatic Brain Injury. Brain Res. 1502, 1–10. 10.1016/j.brainres.2013.01.037 23370001

[B76] Machado F. S. Johndrow J. E. Esper L. Dias A. Bafica A. Serhan C. N. (2006). Anti-inflammatory Actions of Lipoxin A4 and Aspirin-Triggered Lipoxin Are SOCS-2 Dependent. Nat. Med. 12 (3), 330–334. 10.1038/nm1355 16415877

[B77] Maddox J. F. Serhan C. N. (1996). Lipoxin A4 and B4 Are Potent Stimuli for Human Monocyte Migration and Adhesion: Selective Inactivation by Dehydrogenation and Reduction. J. Exp. Med. 183 (1), 137–146. 10.1084/jem.183.1.137 8551217 PMC2192402

[B78] Maderna P. Godson C. (2009). Lipoxins: Resolutionary Road. Br. J. Pharmacol. 158 (4), 947–959. 10.1111/j.1476-5381.2009.00386.x 19785661 PMC2785518

[B79] Mai J. Liu W. Fang Y. Zhang S. Qiu Q. Yang Y. (2018). The Atheroprotective Role of Lipoxin A4 Prevents oxLDL-Induced Apoptotic Signaling in Macrophages via JNK Pathway. Atherosclerosis 278, 259–268. 10.1016/j.atherosclerosis.2018.09.025 30340110

[B80] Marchand F. Perretti M. McMahon S. B. (2005). Role of the Immune System in Chronic Pain. Nat. Rev. Neurosci. 6 (7), 521–532. 10.1038/nrn1700 15995723

[B81] Marcheselli V. L. Hong S. Lukiw W. J. Tian X. H. Gronert K. Musto A. (2003). Novel Docosanoids Inhibit Brain Ischemia-Reperfusion-Mediated Leukocyte Infiltration and Pro-inflammatory Gene Expression. J. Biol. Chem. 278 (44), 43807–43817. 10.1074/jbc.M305841200 12923200

[B82] Martini A. C. Berta T. Forner S. Chen G. Bento A. F. Ji R. R. (2016). Lipoxin A4 Inhibits Microglial Activation and Reduces Neuroinflammation and Neuropathic Pain after Spinal Cord Hemisection. J. Neuroinflammation 13 (1), 75. 10.1186/s12974-016-0540-8 27059991 PMC4826542

[B83] McGeer P. L. Schulzer M. McGeer E. G. (1996). Arthritis and Anti-inflammatory Agents as Possible Protective Factors for Alzheimer's Disease: a Review of 17 Epidemiologic Studies. Neurology 47 (2), 425–432. 10.1212/wnl.47.2.425 8757015

[B84] Medeiros R. Kitazawa M. Passos G. F. Baglietto-Vargas D. Cheng D. Cribbs D. H. (2013). Aspirin-triggered Lipoxin A4 Stimulates Alternative Activation of Microglia and Reduces Alzheimer Disease-like Pathology in Mice. Am. J. Pathol. 182 (5), 1780–1789. 10.1016/j.ajpath.2013.01.051 23506847 PMC3644736

[B85] Mészáros Á. Molnár K. Nógrádi B. Hernádi Z. Nyúl-Tóth Á. Wilhelm I. (2020). Neurovascular Inflammaging in Health and Disease. Cells 9, 1614. 10.3390/cells9071614 PMC7407516 32635451

[B86] Miao G. S. Liu Z. H. Wei S. X. Luo J. G. Fu Z. J. Sun T. (2015). Lipoxin A4 Attenuates Radicular Pain Possibly by Inhibiting Spinal ERK, JNK and NF-κB/p65 and Cytokine Signals, but Not P38, in a Rat Model of Non-compressive Lumbar Disc Herniation. Neuroscience 300, 10–18. 10.1016/j.neuroscience.2015.04.060 25943485

[B87] Mizuma A. Yenari M. A. (2017). Anti-Inflammatory Targets for the Treatment of Reperfusion Injury in Stroke. Front. Neurol. 8, 467. 10.3389/fneur.2017.00467 28936196 PMC5594066

[B88] Moretti L. Cristofori I. Weaver S. M. Chau A. Portelli J. N. Grafman J. (2012). Cognitive Decline in Older Adults with a History of Traumatic Brain Injury. Lancet Neurol. 11 (12), 1103–1112. 10.1016/S1474-4422(12)70226-0 23153408

[B89] Norel X. Brink C. (2004). The Quest for New Cysteinyl-Leukotriene and Lipoxin Receptors: Recent Clues. Pharmacol. Ther. 103 (1), 81–94. 10.1016/j.pharmthera.2004.05.003 15251228

[B90] Pamplona F. A. Ferreira J. Menezes de Lima O. Jr Duarte F. S. Bento A. F. Forner S. (2012). Anti-inflammatory Lipoxin A4 Is an Endogenous Allosteric Enhancer of CB1 Cannabinoid Receptor. Proc. Natl. Acad. Sci. U S A. 109 (51), 21134–21139. 10.1073/pnas.1202906109 23150578 PMC3529012

[B91] Peritore A. F. Crupi R. Scuto M. Gugliandolo E. Siracusa R. Impellizzeri D. (2020). The Role of Annexin A1 and Formyl Peptide Receptor 2/3 Signaling in Chronic Corticosterone-Induced Depression-like Behaviors and Impairment in Hippocampal-dependent Memory. CNS Neurol. Disord. Drug Targets 19 (1), 27–43. 10.2174/1871527319666200107094732 31914916

[B92] Petri M. H. Laguna-Fernandez A. Arnardottir H. Wheelock C. E. Perretti M. Hansson G. K. (2017). Aspirin-triggered Lipoxin A4 Inhibits Atherosclerosis Progression in Apolipoprotein E-/- Mice. Br. J. Pharmacol. 174 (22), 4043–4054. 10.1111/bph.13707 28071789 PMC5659998

[B93] Prieto P. Cuenca J. Través P. G. Fernández-Velasco M. Martín-Sanz P. Boscá L. (2010). Lipoxin A4 Impairment of Apoptotic Signaling in Macrophages: Implication of the PI3K/Akt and the ERK/Nrf-2 Defense Pathways. Cell Death Differ 17, 1179–1188. 10.1038/cdd.2009.220 20094061

[B94] Prieto P. Rosales-Mendoza C. E. Terrón V. Toledano V. Cuadrado A. López-Collazo E. (2015). Activation of Autophagy in Macrophages by Pro-resolving Lipid Mediators. Autophagy 11 (10), 1729–1744. 10.1080/15548627.2015.1078958 26506892 PMC4824594

[B95] Rajkovic O. Potjewyd G. Pinteaux E. (2018). Regenerative Medicine Therapies for Targeting Neuroinflammation after Stroke. Front. Neurol. 9, 734. 10.3389/fneur.2018.00734 30233484 PMC6129611

[B96] Ransohoff R. M. (2016). How Neuroinflammation Contributes to Neurodegeneration. Science 353, 777–783. 10.1126/science.aag2590 27540165

[B97] Romano M. Maddox J. F. Serhan C. N. (1996). Activation of Human Monocytes and the Acute Monocytic Leukemia Cell Line (THP-1) by Lipoxins Involves Unique Signaling Pathways for Lipoxin A4 versus Lipoxin B4: Evidence for Differential Ca2+ Mobilization. J. Immunol. 157 (5), 2149–2154. 8757340

[B98] Sandsmark D. K. Elliott J. E. Lim M. M. (2017). Sleep-Wake Disturbances after Traumatic Brain Injury: Synthesis of Human and Animal Studies. Sleep 40, 40. 10.1093/sleep/zsx044 PMC6251652 28329120

[B99] Schaldach C. M. Riby J. Bjeldanes L. F. (1999). Lipoxin A4: a New Class of Ligand for the Ah Receptor. Biochemistry 38 (23), 7594–7600. 10.1021/bi982861e 10360957

[B100] Serhan C. N. Fiore S. Levy B. D. (1994). Cell-cell Interactions in Lipoxin Generation and Characterization of Lipoxin A4 Receptors. Ann. N. Y Acad. Sci. 744, 166–180. 10.1111/j.1749-6632.1994.tb52734.x 7825838

[B101] Serhan C. N. Hamberg M. Samuelsson B. (1984). Lipoxins: Novel Series of Biologically Active Compounds Formed from Arachidonic Acid in Human Leukocytes. Proc. Natl. Acad. Sci. U S A. 81 (17), 5335–5339. 10.1073/pnas.81.17.5335 6089195 PMC391698

[B102] Serhan C. N. (2014). Pro-resolving Lipid Mediators Are Leads for Resolution Physiology. Nature 510, 92–101. 10.1038/nature13479 24899309 PMC4263681

[B103] Serhan C. N. Sheppard K. A. (1990). Lipoxin Formation during Human Neutrophil-Platelet Interactions. Evidence for the Transformation of Leukotriene A4 by Platelet 12-lipoxygenase *In Vitro* . J. Clin. Invest. 85 (3), 772–780. 10.1172/JCI114503 2155925 PMC296494

[B104] Shearer G. C. Walker R. E. (2018). An Overview of the Biologic Effects of omega-6 Oxylipins in Humans. Prostaglandins Leukot. Essent. Fatty Acids 137, 26–38. 10.1016/j.plefa.2018.06.005 30293594

[B105] Shryock N. McBerry C. Salazar Gonzalez R. M. Janes S. Costa F. T. Aliberti J. (2013). Lipoxin A₄ and 15-Epi-Lipoxin A₄ Protect against Experimental Cerebral Malaria by Inhibiting IL-12/IFN-γ in the Brain. PLoS One 8 (4), e61882. 10.1371/journal.pone.0061882 23613965 PMC3628580

[B106] Smith H. K. Gil C. D. Oliani S. M. Gavins F. N. (2015). Targeting Formyl Peptide Receptor 2 Reduces Leukocyte-Endothelial Interactions in a Murine Model of Stroke. FASEB J. 29 (5), 2161–2171. 10.1096/fj.14-263160 25690650

[B107] Sobrado M. Pereira M. P. Ballesteros I. Hurtado O. Fernández-López D. Pradillo J. M. (2009). Synthesis of Lipoxin A4 by 5-lipoxygenase Mediates PPARgamma-dependent, Neuroprotective Effects of Rosiglitazone in Experimental Stroke. J. Neurosci. 29 (12), 3875–3884. 10.1523/JNEUROSCI.5529-08.2009 19321784 PMC6665034

[B108] Song Y. Yang Y. Cui Y. Gao J. Wang K. Cui J. (2019). Lipoxin A4 Methyl Ester Reduces Early Brain Injury by Inhibition of the Nuclear Factor Kappa B (NF-κb)-dependent Matrix Metallopeptidase 9 (MMP-9) Pathway in a Rat Model of Intracerebral Hemorrhage. Med. Sci. Monit. 25, 1838–1847. 10.12659/MSM.915119 30855024 PMC6423737

[B109] Souza M. C. Pádua T. A. Torres N. D. Souza Costa M. F. Candéa A. P. Maramaldo T. (2015). Lipoxin A4 Attenuates Endothelial Dysfunction during Experimental Cerebral Malaria. Int. Immunopharmacol 24 (2), 400–407. 10.1016/j.intimp.2014.12.033 25576659

[B110] Sun T. Yu E. Yu L. Luo J. Li H. Fu Z. (2012). LipoxinA(4) Induced Antinociception and Decreased Expression of NF-Κb and Pro-inflammatory Cytokines after Chronic Dorsal Root Ganglia Compression in Rats. Eur. J. Pain 16 (1), 18–27. 10.1016/j.ejpain.2011.05.005 21658981

[B111] Svensson C. I. Zattoni M. Serhan C. N. (2007). Lipoxins and Aspirin-Triggered Lipoxin Inhibit Inflammatory Pain Processing. J. Exp. Med. 204 (2), 245–252. 10.1084/jem.20061826 17242163 PMC2118737

[B112] Szczuko M. Kotlęga D. Palma J. Zembroń-Łacny A. Tylutka A. Gołąb-Janowska M. (2020). Lipoxins, RevD1 and 9, 13 HODE as the Most Important Derivatives after an Early Incident of Ischemic Stroke. Sci. Rep. 10 (1), 12849. 10.1038/s41598-020-69831-0 32732956 PMC7393087

[B113] Taetzsch T. Levesque S. McGraw C. Brookins S. Luqa R. Bonini M. G. (2015). Redox Regulation of NF-Κb P50 and M1 Polarization in Microglia. Glia 63, 423–440. 10.1002/glia.22762 25331559 PMC4322433

[B114] Takano T. Clish C. B. Gronert K. Petasis N. Serhan C. N. (1998). Neutrophil-mediated Changes in Vascular Permeability Are Inhibited by Topical Application of Aspirin-Triggered 15-Epi-Lipoxin A4 and Novel Lipoxin B4 Stable Analogues. J. Clin. Invest. 101 (4), 819–826. 10.1172/JCI1578 9466977 PMC508630

[B115] Tanga F. Y. Nutile-McMenemy N. DeLeo J. A. (2005). The CNS Role of Toll-like Receptor 4 in Innate Neuroimmunity and Painful Neuropathy. Proc. Natl. Acad. Sci. U S A. 102 (16), 5856–5861. 10.1073/pnas.0501634102 15809417 PMC556308

[B116] Tian Y. Liu M. Mao-Ying Q. L. Liu H. Wang Z. F. Zhang M. T. (2015). Early Single Aspirin-Triggered Lipoxin Blocked Morphine Anti-nociception Tolerance through Inhibiting NALP1 Inflammasome: Involvement of PI3k/Akt Signaling Pathway. Brain Behav. Immun. 50, 63–77. 10.1016/j.bbi.2015.06.016 26162710

[B117] Tiffany H. L. Lavigne M. C. Cui Y. H. Wang J. M. Leto T. L. Gao J. L. (2001). Amyloid-beta Induces Chemotaxis and Oxidant Stress by Acting at Formylpeptide Receptor 2, a G Protein-Coupled Receptor Expressed in Phagocytes and Brain. J. Biol. Chem. 276 (26), 23645–23652. 10.1074/jbc.M101031200 11316806

[B118] Tobin D. M. Roca F. J. Oh S. F. McFarland R. Vickery T. W. Ray J. P. (2012). Host Genotype-specific Therapies Can Optimize the Inflammatory Response to Mycobacterial Infections. Cell. 148 (3), 434–446. 10.1016/j.cell.2011.12.023 22304914 PMC3433720

[B119] Trovato A. Siracusa R. Di Paola R. Scuto M. Fronte V. Koverech G. (2016). Redox Modulation of Cellular Stress Response and Lipoxin A4 Expression by Coriolus Versicolor in Rat Brain: Relevance to Alzheimer's Disease Pathogenesis. Neurotoxicology 53, 350–358. 10.1016/j.neuro.2015.09.012 26433056

[B120] Tylek K. Trojan E. Regulska M. Lacivita E. Leopoldo M. Basta-Kaim A. (2021). Formyl Peptide Receptor 2, as an Important Target for Ligands Triggering the Inflammatory Response Regulation: a Link to Brain Pathology. Pharmacol. Rep. 73, 1004–1019. 10.1007/s43440-021-00271-x 34105114 PMC8413167

[B121] Vasconcelos D. P. Costa M. Amaral I. F. Barbosa M. A. Águas A. P. Barbosa J. N. (2015). Modulation of the Inflammatory Response to Chitosan through M2 Macrophage Polarization Using Pro-resolution Mediators. Biomaterials 37, 116–123. 10.1016/j.biomaterials.2014.10.035 25453942

[B122] Vital S. A. Becker F. Holloway P. M. Russell J. Perretti M. Granger D. N. (2016). Formyl-Peptide Receptor 2/3/Lipoxin A4 Receptor Regulates Neutrophil-Platelet Aggregation and Attenuates Cerebral Inflammation: Impact for Therapy in Cardiovascular Disease. Circulation 133 (22), 2169–2179. 10.1161/CIRCULATIONAHA.115.020633 27154726 PMC4889496

[B123] Wada K. Arita M. Nakajima A. Katayama K. Kudo C. Kamisaki Y. (2006). Leukotriene B4 and Lipoxin A4 Are Regulatory Signals for Neural Stem Cell Proliferation and Differentiation. FASEB J. 20 (11), 1785–1792. 10.1096/fj.06-5809com 16940150

[B124] Wang G. Zhang L. Chen X. Xue X. Guo Q. Liu M. (2016). Formylpeptide Receptors Promote the Migration and Differentiation of Rat Neural Stem Cells. Sci. Rep. 6, 25946. 10.1038/srep25946 27173446 PMC4865803

[B125] Wang X. Miao Z. Xu X. Schultzberg M. Zhao Y. (2021). Reduced Levels of Plasma Lipoxin A4 Are Associated with Post-Stroke Cognitive Impairment. J. Alzheimers Dis. 79 (2), 607–613. 10.3233/JAD-201050 33337374

[B126] Wang X. Zhu M. Hjorth E. Cortés-Toro V. Eyjolfsdottir H. Graff C. (2015). Resolution of Inflammation Is Altered in Alzheimer's Disease. Alzheimers Dement 11 (1), 40–42. 10.1016/j.jalz.2013.12.024 24530025 PMC4275415

[B127] Wang Y. P. Wu Y. Li L. Y. Zheng J. Liu R. G. Zhou J. P. (2011). Aspirin-triggered Lipoxin A4 Attenuates LPS-Induced Pro-inflammatory Responses by Inhibiting Activation of NF-Κb and MAPKs in BV-2 Microglial Cells. J. Neuroinflammation 8, 95. 10.1186/1742-2094-8-95 21831303 PMC3162900

[B128] Wang Z. F. Li Q. Liu S. B. Mi W. L. Hu S. Zhao J. (2014). Aspirin-triggered Lipoxin A4 Attenuates Mechanical Allodynia in Association with Inhibiting Spinal JAK2/STAT3 Signaling in Neuropathic Pain in Rats. Neuroscience 273, 65–78. 10.1016/j.neuroscience.2014.04.052 24836854

[B129] Wilson L. Stewart W. Dams-O'Connor K. Diaz-Arrastia R. Horton L. Menon D. K. (2017). The Chronic and Evolving Neurological Consequences of Traumatic Brain Injury. Lancet Neurol. 16 (10), 813–825. 10.1016/S1474-4422(17)30279-X 28920887 PMC9336016

[B130] Wu H. S. Guo P. P. Jin Z. Li X. Y. Yang X. Ke J. J. (2018). Effects of Lipoxin A4 Pretreatment on Cognitive Function of Aged Rats after Global Cerebral Ischemia Reperfusion. Curr. Med. Sci. 38 (4), 666–671. 10.1007/s11596-018-1928-8 30128876

[B131] Wu J. Ding D. Wang X. Li Q. Sun Y. Li L. (2019a). Regulation of Aquaporin 4 Expression by Lipoxin A4 in Astrocytes Stimulated by Lipopolysaccharide. Cell Immunol 344, 103959. 10.1016/j.cellimm.2019.103959 31383359

[B132] Wu J. Ding D. H. Li Q. Q. Wang X. Y. Sun Y. Y. Li L. J. (2019b). Lipoxin A4 Regulates Lipopolysaccharide-Induced BV2 Microglial Activation and Differentiation via the Notch Signaling Pathway. Front Cel Neurosci 13, 19. 10.3389/fncel.2019.00019 PMC6369213 30778288

[B133] Wu J. Wang A. Min Z. Xiong Y. Yan Q. Zhang J. (2011). Lipoxin A4 Inhibits the Production of Proinflammatory Cytokines Induced by β-amyloid *In Vitro* and *In Vivo* . Biochem. Biophys. Res. Commun. 408, 382–387. 10.1016/j.bbrc.2011.04.013 21501589

[B134] Wu L. Li H. H. Wu Q. Miao S. Liu Z. J. Wu P. (2015). Lipoxin A4 Activates Nrf2 Pathway and Ameliorates Cell Damage in Cultured Cortical Astrocytes Exposed to Oxygen-Glucose Deprivation/Reperfusion Insults. J. Mol. Neurosci. 56 (4), 848–857. 10.1007/s12031-015-0525-6 25702137

[B135] Wu L. Liu Z. J. Miao S. Zou L. B. Cai L. Wu P. (2013). Lipoxin A4 Ameliorates Cerebral Ischaemia/reperfusion Injury through Upregulation of Nuclear Factor Erythroid 2-related Factor 2. Neurol. Res. 35 (9), 968–975. 10.1179/1743132813Y.0000000242 23880501

[B136] Wu L. Miao S. Zou L. B. Wu P. Hao H. Tang K. (2012a). Lipoxin A4 Inhibits 5-lipoxygenase Translocation and Leukotrienes Biosynthesis to Exert a Neuroprotective Effect in Cerebral Ischemia/reperfusion Injury. J. Mol. Neurosci. 48 (1), 185–200. 10.1007/s12031-012-9807-4 22661361

[B137] Wu Y. Wang Y. P. Guo P. Ye X. H. Wang J. Yuan S. Y. (2012b). A Lipoxin A4 Analog Ameliorates Blood-Brain Barrier Dysfunction and Reduces MMP-9 Expression in a Rat Model of Focal Cerebral Ischemia-Reperfusion Injury. J. Mol. Neurosci. 46 (3), 483–491. 10.1007/s12031-011-9620-5 21845429

[B138] Wu Y. Zhai H. Wang Y. Li L. Wu J. Wang F. (2012c). Aspirin-triggered Lipoxin A₄ Attenuates Lipopolysaccharide-Induced Intracellular ROS in BV2 Microglia Cells by Inhibiting the Function of NADPH Oxidase. Neurochem. Res. 37 (8), 1690–1696. 10.1007/s11064-012-0776-3 22552474

[B139] Wu Y. Ye X. H. Guo P. P. Xu S. P. Wang J. Yuan S. Y. (2010). Neuroprotective Effect of Lipoxin A4 Methyl Ester in a Rat Model of Permanent Focal Cerebral Ischemia. J. Mol. Neurosci. 42 (2), 226–234. 10.1007/s12031-010-9355-8 20401639

[B140] Xue M. Yong V. W. (2020). Neuroinflammation in Intracerebral Haemorrhage: Immunotherapies with Potential for Translation. Lancet Neurol. 19 (12), 1023–1032. 10.1016/S1474-4422(20)30364-1 33212054

[B141] Yao C. Yang D. Wan Z. Wang Z. Liu R. Wu Y. (2014). Aspirin-triggered Lipoxin A4 Attenuates Lipopolysaccharide Induced Inflammatory Response in Primary Astrocytes. Int. Immunopharmacol 18 (1), 85–89. 10.1016/j.intimp.2013.10.028 24269179

[B142] Ye R. D. Boulay F. Wang J. M. Dahlgren C. Gerard C. Parmentier M. (2009). International Union of Basic and Clinical Pharmacology. LXXIII. Nomenclature for the Formyl Peptide Receptor (FPR) Family. Pharmacol. Rev. 61, 119–161. 10.1124/pr.109.001578 19498085 PMC2745437

[B143] Ye X. H. Wu Y. Guo P. P. Wang J. Yuan S. Y. Shang Y. (2010). Lipoxin A4 Analogue Protects Brain and Reduces Inflammation in a Rat Model of Focal Cerebral Ischemia Reperfusion. Brain Res. 1323, 174–183. 10.1016/j.brainres.2010.01.079 20138164

[B144] Zhu J. J. Yu B. Y. Fu C. C. He M. Z. Zhu J. H. Chen B. W. (2020). LXA4 Protects against Hypoxic-Ischemic Damage in Neonatal Rats by Reducing the Inflammatory Response via the IκB/NF-Κb Pathway. Int. Immunopharmacol 89 (Pt B), 107095. 10.1016/j.intimp.2020.107095 33096360

[B145] Zhu M. Wang X. Hjorth E. Colas R. A. Schroeder L. Granholm A. C. (2016). Pro-Resolving Lipid Mediators Improve Neuronal Survival and Increase Aβ42 Phagocytosis. Mol. Neurobiol. 53 (4), 2733–2749. 10.1007/s12035-015-9544-0 26650044 PMC4824659

[B146] Zhu M. Wang X. Schultzberg M. Hjorth E. (2015). Differential Regulation of Resolution in Inflammation Induced by Amyloid-Β42 and Lipopolysaccharides in Human Microglia. J. Alzheimers Dis. 43 (4), 1237–1250. 10.3233/JAD-141233 25147114

